# Pulsed‐Laser‐Driven CO_2_ Reduction Reaction for the Control of the Photoluminescence Quantum Yield of Organometallic Gold Nanocomposites

**DOI:** 10.1002/smsc.202300328

**Published:** 2024-04-01

**Authors:** Tahir, Guilherme C. Concas, Mariana Gisbert, Marco Cremona, Fernando Lazaro, Marcelo Eduardo H. Maia da Costa, Suellen D. T. De Barros, Ricardo Q. Aucélio, Tatiana Saint Pierre, José Marcus Godoy, Diogo Mendes, Gino Mariotto, Nicola Daldosso, Francesco Enrichi, Alexandre Cuin, Aldebarã F. Ferreira, Walter M. de Azevedo, Geronimo Perez, Celso SantAnna, Braulio Soares Archanjo, Yordy E. Licea Fonseca, Andre L. Rossi, Francis L. Deepak, Rajwali Khan, Quaid Zaman, Sven Reichenberger, Theo Fromme, Giancarlo Margheri, José R. Sabino, Gabriella Fibbi, Mario Del Rosso, Anastasia Chillà, Francesca Margheri, Anna Laurenzana, Tommaso Del Rosso

**Affiliations:** ^1^ Department of Physics Pontifical Catholic University of Rio de Janeiro Rua Marquês de São Vicente 225 22451‐900 Gávea Rio de Janeiro Brazil; ^2^ Department of Chemistry Pontifical Catholic University of Rio de Janeiro Rua Marquês de São Vicente 225 22451‐900 Gávea Rio de Janeiro Brazil; ^3^ Department of Engineering for Innovation Medicine University of Verona Strada le Grazie 15 37134 Verona Italy; ^4^ Departamento de Química Instituto de Ciências Exatas Universidade Federal de Juiz de Fora Juiz de Fora MG 36036‐900 Brazil; ^5^ Department of Fundamental Chemistry Federal University of Pernambuco Av. Jorn. Aníbal Fernandes, s/n ‐ Cidade Universitária 50740‐560 Recife PE Brazil; ^6^ Departamento de Engenharia Mecânica Universidade Federal Fluminense Rua Passo da Pátria, 156 Campus da Praia Vermelha 24210‐240 Niterói Brazil; ^7^ National Institute of Metrology Quality and Technology (Inmetro) Av. Nossa Senhor das Graças 50 25250‐020 Duque de Caxias RJ Brazil; ^8^ Department of Production GlaxoSmithKline SpA (GSK) 9911 Beldward Campus Dr. 20850 Rockville MD USA; ^9^ Centro Brasileiro de Pesquisas Físicas (CBPF) R. Dr. Xavier Sigaud 150 22290‐180 Urca Rio de Janeiro Brazil; ^10^ International Iberian Nanotechnology Laboratory Nanostructured Materials Group Avenida Mestre Jose Veiga Braga 4715‐330 Portugal; ^11^ Department of Physics United Arab Emirates University Sheik Khalifa Bin Zayed Street Al‐Ain 15551 United Arab Emirates; ^12^ Department of Physics University of Buner Main Sowari Bazzar 17290 Buner Pakistan; ^13^ Department of Technical Chemistry I University of Duisburg‐Essen Universitätsstraße 5 45141 Essen Germany; ^14^ Center for Nanointegration Duisburg‐Essen (CENIDE) University of Duisburg‐Essen Carl‐Benz‐Straße 199 47057 Duisburg Germany; ^15^ Istituto dei Sistemi Complessi Sezione di Sesto Fiorentino (I.S.C – CNR) Via Madonna delPiano 10 50019 Sesto Fiorentino Italy; ^16^ Institute of Physics Federal University of Goias Av. Esperança 74690‐970 Goiânia Brazil; ^17^ Department of Experimental and Clinical Biomedical Sciences University of Florence Viale Morgagni 50 50134 Florence Italy

**Keywords:** CO_2_ reduction reaction, laser synthesis and processing of colloids in water, organometallic colloidal nanocomposites, photoluminescence quantum yield

## Abstract

Over the last decade, the CO_2_ reduction reaction (CO_2_RR) has been increasingly exploited for the synthesis of high‐value raw materials in gaseous or liquid form, although no examples of CO_2_ fixation in nanoparticle systems have been demonstrated. Herein, CO_2_ fixation into solid nanomaterials by laser synthesis and processing of gold colloids in water, traditionally considered a green approach leading to ligand‐free nanoparticles without the formation of by‐products, is reported. If carbon monoxide‐rich gold nanoparticles are observable even after synthesis in deionized water, the presence of CO_2_ derivatives in alkaline water environment leads to *C*_2_ and *C*_3_ coupling with the production of carboxylic acids as a typical CO_2_RR fingerprint. While laser processing of preformed gold colloids is selective for *C*_2_ coupling, both *C*_2_ and *C*_3_ coupling to lactic acid are observed during pulsed laser ablation of a gold target. In the latter case, it is demonstrated that it is possible to synthesize photoluminescent organometallic nanocomposites in the blue spectral region with a quantum yield of about 20% under adequate experimental conditions. In this research, new pathways are offered to be explored in energetics, photonics, catalysis, and synthesis at the nanoscale.

## Graphical abstract

Pulsed‐laser‐driven CO_2_ reduction reaction allows the synthesis of carbon‐monoxide‐rich gold nanoparticles and organometallic nanocomposites composed by gold nanoclusters and carboxylic salts, characterized by a photoluminescent quantum yield of 20% in the visible (blue) region.

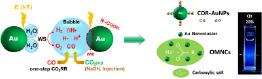

## 1 Introduction

Several efforts have been directed toward the development of negative emission technologies in the last decade, where CO_2_ capture, storage, and modification into value‐added chemicals is considered to have an energetic value.^[^ ^1^ ^]^ The chemical reduction of CO_2_ into gases or fuels has been mainly demonstrated by the use of electrochemical cells at low potential regimes, although thermochemical and photochemical cells employing different nanostructured interfaces have also been developed.^[^ ^2^, ^3^, ^4^, ^5^, ^6^, ^7^, ^8^, ^9^ ^]^ Reduction of CO_2_ to solid carbon is now considered one of the most valuable routes to a permanent storage solution.^[^ ^10^, ^11^ ^]^ In fact, the CO_2_ reduction reaction (CO_2_RR) in volatile species such as methane, carbon monoxide, or organic solvents is not a definite route concerning environmental pollution, and the unwanted release of such species into the environment still represents a potentially serious hazard. Thus, intensive research has been devoted to the development of alternative technologies capable of further converting more volatile species into less‐volatile functional materials.

Over the last five decades, pulsed lasers have created many new technological possibilities, from laser cutting, drilling, welding, and engraving to 3D printing^[^ ^12^, ^13^ ^]^ and the laser synthesis or processing of colloids (LSPC).^[^ ^14^ ^]^ The LSPC method includes both nanoparticle synthesis by pulsed laser ablation in a liquid (PLAL), or laser irradiation of preformed nanoparticles.^[^ ^15^, ^16^, ^17^ ^]^ In the latter case, different physicochemical mechanisms can be triggered considering gold nanoparticles (AuNPs) in water, depending on the laser pulse wavelength, fluence, temporal duration and repetition rate. For example, laser pulses at the wavelength of 532 nm can induce a so‐called nanoparticle laser fragmentation in liquid (LFL) if the fluence is higher than ≈0.5 J cm^−2^,^[^ ^14^ ^]^ leading to a metal nanoparticle phase explosion and the recombination of the gold clusters into small nanoparticles averaging about ≈3 nm.^[^ ^17^, ^18^ ^]^

In addition to PLAL comprising a scalable synthesis method for high‐purity nanoparticles, persistent microbubbles containing oxygen and hydrogen and, in some cases, hydrogen peroxide, have also been observed.^[^ ^15^, ^19^ ^]^ It has been discussed that permanent gases originate from thermally induced water splitting (WS), due to direct contact with the water layer above the hot plasma plume that forms within the laser‐excited target region during the first few nanoseconds, following the absorption of the high‐intensity incident laser pulse.^[^ ^20^ ^]^ Apart from the direct production of hydrogen and oxygen during the laser ablation of a solid target in water, colloidal nanoparticles have also been synthesized in the past by reducing metal salts applying high‐intensity laser pulses (typically short ps to fs pulses).^[^ ^21^ ^]^ As demonstrated by the Tibbetts group, the production of radicals, such as (H•/OH•) and (H+/OH−), is key to understanding their respective redox reactions.^[^ ^22^ ^]^

Moreover, the production of molecular hydrogen has also been observed during the pulsed laser irradiation of preformed colloidal AuNPs dispersions, where the excited metal electrons can trigger a cascade ionization and water molecule breakdown. In this case, appropriate pulse fluence values and metal species concentrations encompass the key factors controlling the gas rate and volume produced during irradiation.^[^ ^23^, ^24^, ^25^ ^]^

More recently, the CO_2_RR has also been observed when performing laser reduction in liquids employing ultrashort, high‐intensity laser pulses in water containing CO_2_ without the presence of AuNPs. In this case, the authors reported the formation of CO and small amounts of CH_4_.^[^ ^26^ ^]^ In contrast, the addition of a transition metal target, such as gold, can further reduce the Gibbs free energy for the CO_2_RR, due to higher local temperatures reached near the metal target during the process (10^4^ K),^[^ ^27^ ^]^ while also introducing other additional reactive species, such as molecular hydrogen (H_2_)^[^ ^2^, ^19^ ^]^ and solvated electrons (e^−^). These may, in turn, further promote CO_2_ activation and the stabilization of intermediate reaction products, eventually increasing the CO_2_RR to CO.^[^ ^28^, ^29^ ^]^ However, the presence of CO_2_ in the liquid environment has received poor attention in previous studies. In fact, carbonates and organic material have been detected during gold PLAL in water when ablation was performed at high pH values.^[^ ^30^, ^31^, ^32^ ^]^ Some authors^[^ ^30^ ^]^ have previously suggested that oxocarbons may originate from nanomaterial contamination when in contact with the atmosphere, although the origin of the organic material has not yet been identified. Even less attention has been paid to the potential chemical reactions involved in the pulsed laser processing of preformed colloids, such as AuNPs fusion or fragmentation, with the literature mainly devoted to the discrimination of the threshold fluence of these processes, and the morphology and charge of the resulting nanomaterial.^[^ ^14^, ^17^, ^33^ ^]^ Both PLAL and LFL in water are traditionally considered as obeying an important green chemistry rule, i.e., the absence of by‐products, which is why the resulting nanoparticles are commonly defined as ligand free in most literature reports.^[^ ^15^, ^34^, ^35^ ^]^

A systematic investigation including negative control experiments with inert gases at the gas‐liquid interface was carried herein to evaluate the possibility of inducing the CO_2_RR by gold colloids laser synthesis and processing.^[^ ^14^ ^]^ Alongside CO production, the pulsed‐laser‐driven CO_2_RR was confirmed to allow the control of both *C*_2_ and *C*_3_ coupling processes. Furthermore, CO_2_ fixation leads to organometallic photoluminescent nanocomposites obtained by the complexation or nucleation of organic products with AuNPs.

In this context, Ziefuß et al. recently demonstrated that LFL in water allows for the synthesis of fully inorganic gold nanoclusters (AuNCs) characterized by a quantum yield (QY) of about 2% for visible (blue) emission.^[^ ^36^ ^]^ A common way to increase the stability and the QY of AuNCs to values between 10% and 60% is to manipulate the electronic environment by adding ligands that act as electron donors.^[^ ^37^, ^38^, ^39^, ^40^, ^41^ ^]^ Therefore, the possibility of carrying out the CO_2_RR in situ during PLAL of gold targets in water is an intriguing perspective for the production of photoluminescent AuNCs where stability and QY enhancement can be controlled by the same organic ligands produced during the reaction.

## 2 Results and Discussion

### 2.1 AuNPs Size Control

The PLAL was performed for a total of 6 h by irradiating the same gold target point with laser pulses at different wavelengths, namely 1064 nm (*ω*) or 532 nm (2*ω*), as well as simultaneous irradiation employing pulses at both 1064 and 532 nm (*ω *+ 2*ω*). The experimental setup used for the synthesis is depicted in Figure S1, Supporting Information, while the laser parameter values of the different PLAL configurations are reported in detail in Table S1, Supporting Information.

**Figure 1:**
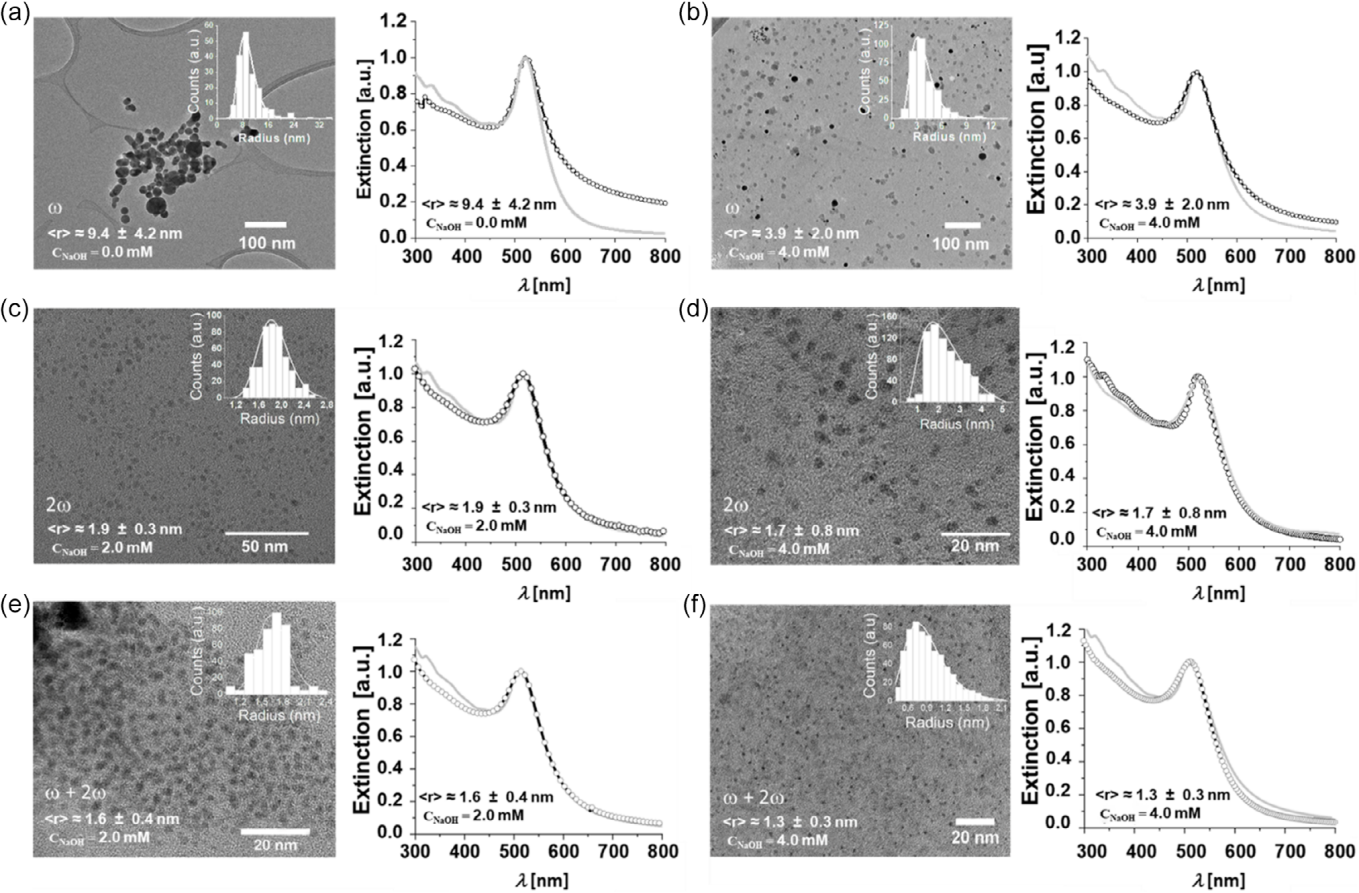
a–f) TEM images of AuNPs synthesized by PLAL in water in equilibrium with the air environment employing laser pulses at different frequencies and different *C*_NaOH_ values. a,b) Laser pulses at the *ω* frequency in deionized water or in water with a *C*_NaOH_ value of 4.0 mmol L^−1^. c,d) Laser pulses at the 2*ω* frequency in water with *C*_NaOH_ values of 2.0 or 4.0 mmol L^−1^. e,f) Simultaneous laser pulse irradiation at both the *ω* and 2*ω* frequencies (*ω* + 2*ω*) in water with *C*_NaOH_ values of 2.0 or 4.0 mmol L^−1^. The insets depict the experimental statistical size (radius) distribution. The UV–vis extinction spectra of the corresponding colloidal dispersion are represented on the right side of the TEM images. The grey lines represent the Mie fit obtained in the dipolar approximation applying the experimental size distributions.

**Figure** **1**a–f presents the AuNPs transmission electron microscopy (TEM) images as a function of the sodium hydroxide (NaOH) concentration in the aqueous solution (*c*_NaOH_), and laser pulse frequencies. As indicated in Table S2, Supporting Information, both pH (i.e., *c*_NaOH_) and laser pulse frequency control the statistical average nanoparticle dimensions. In the case of laser pulses at the *ω* frequency, the introduction of NaOH is responsible for the evident differences observed concerning statistical nanoparticle size distribution. Without the addition of NaOH (Figure 1a), the AuNPs present primary and secondary nanoparticles with average diameters of about 20 and 45 nm,^[^ ^42^ ^]^ respectively, while the broadening of the extinction spectra up to wavelengths of about 800 nm is attributed to the low pH and ionic force of deionized water,^[^ ^30^ ^]^ which provokes interparticle dipolar interaction, large coalescence, and agglomeration.^[^ ^43^, ^44^ ^]^ When NaOH is introduced into the ablation environment, the average nanoparticle diameter is reduced to ≈8 nm, the extinction spectra are narrower, and the size distribution becomes monomodal, without the detection of a significant number of secondary nanoparticles (Figure 1b). This agrees with literature results concerning AuNPs synthesized by PLAL in water in basic environments or those containing highly diluted electrolytes,^[^ ^30^, ^45^ ^]^ where primary nanoparticle coalescence is inhibited during main cavitation bubble dynamics.^[^ ^42^, ^46^ ^]^

In contrast, applying pulses at 532 nm leads to monomodal distributions with average AuNPs dimensions well below 10 nm (Figure 1c–f), down to an average diameter *ϕ* of about 2.6 nm for *c*_NaOH_ = 4.0 mmol L^−1^ in the *ω *+ 2*ω* configuration (Figure 1f). As indicated by the theoretical calculations reported in Figure S2, Supporting Information, although the continuous formation of new AuNPs is observed throughout the irradiation process, the fluence of the laser pulse passing through the preformed nanoparticle column before reaching the target is, in this case, reduced to values below ≈1.5 J cm^−2^ during laser processing, leading to an overlap between the synchronous target ablation and LFL.^[^ ^14^, ^33^, ^36^ ^]^

The size and stability of AuNPs synthesized by PLAL in water media containing hydroxides are commonly associated to the pH of the aqueous solution, which controls electrostatic nanoparticle repulsion, modulating AuO^−^ and AuOH group ratios on the nanoparticle surface.^[^ ^30^, ^36^ ^]^ The results reported in Table S2, Supporting Information, confirm this trend, where nanoparticle sizes decrease at higher pH values. The observed particle size and higher yield of smaller AuNPs (i.e., *ϕ *< 3 nm) with increasing pH values are also in agreement with the recent study published by Ziefuß et al., who described the synergism between anion adsorption and high pH values in electrostatically stabilized AuNCs.^[^ ^47^ ^]^

In addition to morphological characterizations, we further investigated the organic content in the colloidal dispersions of AuNPs by determining the parameter Δ*TC*, which represents the total carbon (TC) difference between samples obtained after laser pulse irradiation with (PLAL) or without (laser reduction in liquid) the gold target (Figure S3, Supporting Information), but with the same value of *c*_NaOH_. As indicated in Table S2, Supporting Information, the results indicate that the final TC value following laser incidence on the NaOH solution (*TC*_0_) is lower when the gold target is absent, suggesting that chemical reactions involving aqueous carbon dioxide (CO_2aq_) species occur during gold target PLAL.

The presence of by‐products originating from the PLAL process different from bare gold nanoparticles is evident in Figure 1b,f. In the first case, spots presenting a lower contrast are observed, corresponding to small nanoparticles with an average diameter of about 5 nm, which rapidly volatilize when attempting to capture higher‐magnification images (higher electron current density). In the second case, the by‐products form an extended medium in which ultrasmall gold nanoparticles are embedded.

### 2.2 Products of the Pulsed‐Laser‐Driven CO_2_RR on Gold

**Figure 2:**
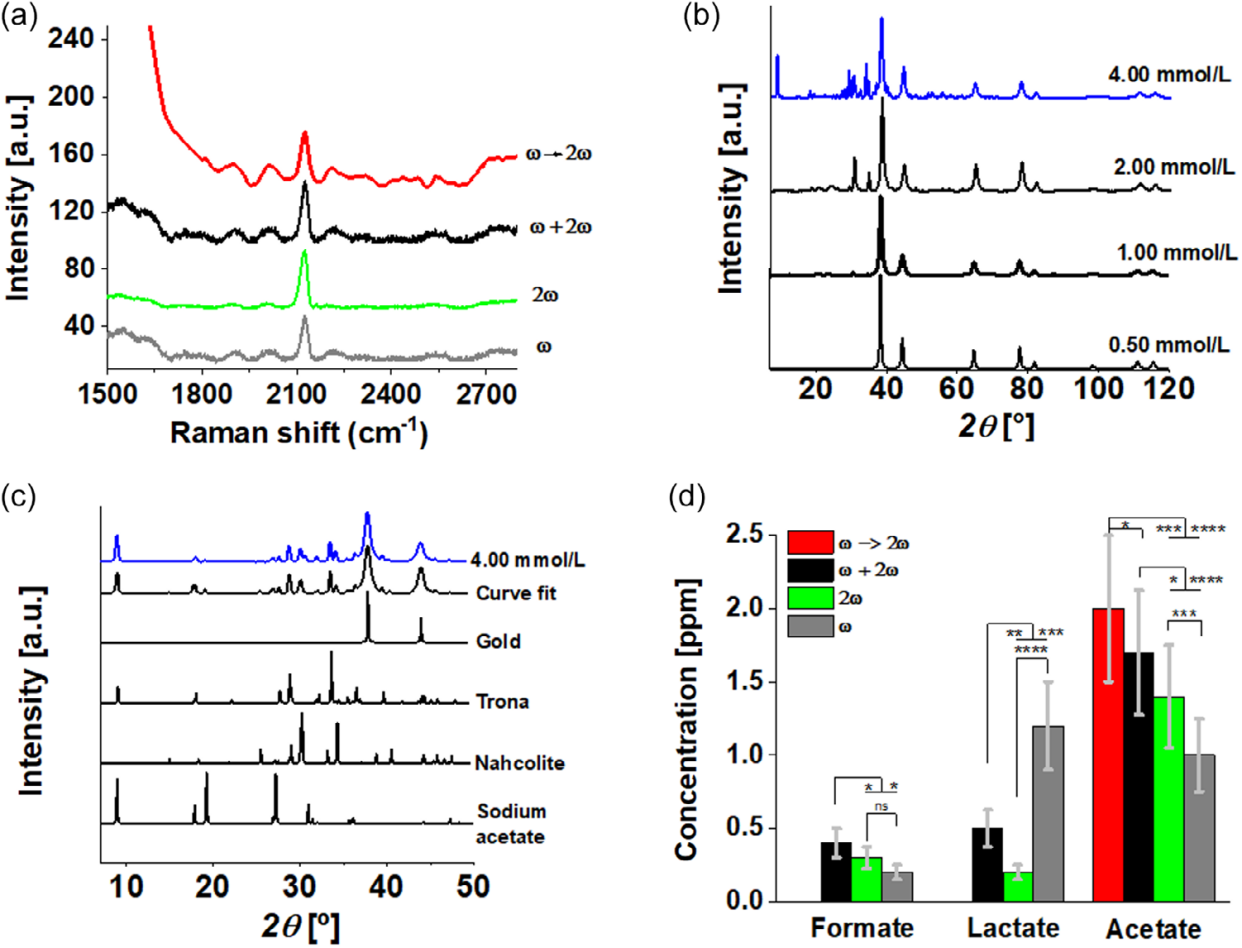
a) SERS spectra of the nanomaterial synthesized in water at *C*_NaOH_  = 2.0 mmol L^−1^ in equilibrium with air by PLAL at 532 nm (2*ω*, green line), 1064 nm (*ω*, grey line), or 532 nm and 1064 nm (*ω* + 2*ω*, black line), and by LFL (*ω* → 2*ω*, red line). The excitation wavelength in the SERS measurements was 638 nm. Some of the spectra were shifted along the vertical axes for better visualization. b) XRD patterns of the crystalline materials synthesized by PLAL at the 2*ω* frequency in water at different *C*_NaOH_ values in equilibrium with air. c) Theoretical fit of the experimental XRD spectra for *C*_NaOH_ = 4.0 mmol L^−1^ (blue line). Several crystalline organic species were detected alongside the typical gold phase diffraction pattern, such as nahcolite, trona, and sodium acetate. d) Carboxylic ion concentrations determined by ion chromatography on the transparent nanomaterial diffusing through the pores of a dialysis membrane with a molecular weight cutoff of 3.5 kD. The pristine nanomaterial was synthesized by LFL (red columns) or PLAL with laser pulses at the *ω* frequency (grey columns), 2*ω* frequency (green columns), and *ω* + 2*ω* (black columns) in water at *C*_NaOH_ = 4 mmol L^−1^ and in equilibrium with air. As reported in Table S2, Supporting Information, the total carbon of the samples (*TC*_0_ + Δ*TC*) was about 35 ppm. Data are presented as the means ± SD (*n* = 5) (ns, not significant *p < 0.5, **p < 0.01, ***p < 0.001, and ****p < 0.0001).

A detailed analysis of the nanomaterial produced by the pulsed‐laser‐driven CO_2_RR was performed by surface‐enhanced Raman scattering (SERS) to demonstrate that Au–CO ligands are present on the surface of the nanoparticles before their exposure to the air. We first examined the SERS spectra of the AuNPs subjected to LSPC at the water–air interface in the presence of NaOH. The results, presented in **Figure** **2**a, indicate that the Au–CO signal, centered at about 2120 cm^−1^, appears regardless of experimental condition and configuration. Figure S4, Supporting Information, displays the SERS spectra of AuNPs synthesized at different water–gas interfaces without NaOH, namely in pure argon (AuNPs_AR_), air (AuNPs_Air_), nitrogen (AuNP_N2_), and a 99% argon and 1% CO_2_ (${\text{AuNP}}_{CO_{2}}$) mixture. It is interesting to note that, in this case, the gold carbonyl signal is absent from all spectra. The SERS signal depends on the LSPR band of the spatial region excited by the laser radiation, which is quenched by the fast and massive agglomeration that takes place during the SERS analysis sample preparation (see Experimental Section). For example, Figure S4, Supporting Information, also reports the Raman spectra of the lyophilized powders synthesized with *c*_NaOH_ = 2.0 mmol L^−1^, in which the gold carbonyl signal is absent, suggesting that AuNPs precipitation must be avoided during the SERS substrate deposition process. To confirm this observation, we added NaOH to the colloidal AuNPs dispersion following syntheses at different water‐gas interfaces to improve stability, and repeated the SERS measurements. As noted in Figure S4, Supporting Information, both AuNPs_Air_ and AuNP_CO2_ present a gold‐carbonyl signal, confirming that Au–CO is not absorbed onto the NPs surface when in contact with the atmosphere, but is instead generated during the LSPC in the presence of CO_2aq_. These results are particularly significant, as gold‐carbonyl species are detected even at very low CO_2aq_ concentrations, such as in the case of deionized water in equilibrium with air (TC < 1 ppm). The results, supported by the recent observation concerning the laser reduction of CO_2_ to CO in water by Yan et al.,^[^ ^26^ ^]^ confirm that the CO_2_RR to CO transformation during the gold target PLAL in water is responsible for the detection of the gold‐carbonyl line, and that the concept of a ligand‐free nanoparticle cannot be applied when a gold target PLAL is performed at the water–air interface.

To investigate the pulsed‐laser‐driven CO_2_RR characteristics, we studied the *c*_NaOH_ dependence of the X‐Ray diffraction (XRD) pattern of the nanomaterial produced in water in equilibrium with air. Figure 2b indicates that several Bragg reflections begin to appear below the most intense gold phase peak at 38.2 (2Θ) at *c*_NaOH_ > 2.0 mmol L^−1^, alongside traditional peaks associated with crystalline gold, linked with a preferential growth along the (111) plane.^[^ ^48^ ^]^ Vice versa, the XRD pattern at a lower NaOH concentration exhibited the gold phase only. Applying the procedure described in Experimental Section (using the Joint Committee on Powder Diffraction Standards, JCPDS, Supporting Information), a fit was performed to the nanomaterial diffractogram obtained at a *c*_NaOH_ of 4.0 mmol L^−1^ and laser pulses at a 2*ω* frequency,^[^ ^49^ ^]^ yielding the results presented in Figure 2c. Qualitative and quantitative XRD analyses demonstrated the formation of typical products of both CO_2_RR and NaOH‐based CO_2_ capture and storage, such as trona (Na_3_(CO_3_)(HCO_3_).2(H_2_O)), nahcolite (NaHCO_3_), and sodium acetate (NaCH_3_COO) at 49.9%, 39.2%, and 10.9%, respectively.^[^ ^50^, ^51^, ^52^, ^53^ ^]^ These percentages are relative to the organic material mass detected in the crystalline phase, and therefore do not consider possible amorphous CO_2_RR products or the organic material fraction volatilized during the powder preparation for the analysis.^[^ ^54^ ^]^

To confirm the XRD results and further investigate the effect of the experimental laser irradiation parameters, an ion chromatography (IC) analysis was performed on the portion of the material diffusing through the pores of a dialysis membrane with a molecular weight cutoff of 3.5 kD, corresponding to an average diameter of 1.8 nm for gold. Table S3, Supporting Information, and Figure 2d display the determined organic acid concentrations obtained from pristine samples synthesized in water at equilibrium with air (*c*_NaOH_ = 4.0 mmol L^−1^, TC = (TC_0 _+ ΔTC) = 35 ppm) by LFL or PLAL applying laser pulses at different frequencies. As organic carboxylic acids tend to be slightly volatile, a fraction evaporates during sample concentration employing mild thermal heating, so that the concentration determined by IC represents a lower value than the actual one.^[^ ^54^ ^]^

Only a *C*_2_ coupling to acetic acid is observed following LFL. In this case, the *C*_2_ coupling efficiency, defined as the ratio between the concentration of *C*_2_ products and TC (TC = 35 ppm), is of about 10%. In contrast, when PLAL is performed, the results highlight the presence of formate, acetate, and lactate ions, as indicated in the elution curve presented in Figure S5, Supporting Information. It is interesting to note that the *C*_3_/*C*_2_ coupling ratio varies depending on the laser pulse frequency used to perform the PLAL (Table S3, Supporting Information). This parameter has a minimum value of 0.14 when 2*ω* pulses are used, increasing to 0.29 during simultaneous irradiation with *ω* and 2*ω* pulses, and is higher than 1 when PLAL is performed by infrared laser pulses. We conclude that a selective *C*_2_ coupling to acetic acid is obtained by LFL (at least at the experimented fluence), while the *C*_3_ coupling is a distinct process associated to target ablation, with the highest conversion efficiency of ≈3% obtained by pulses applied at the *ω* frequency (pure PLAL process without fragmentation). Concerning the results presented in Figure S2, Supporting Information, a qualitative correlation was noted between the rate of new nanoparticle production during PLAL, proportional to the extinction spectra slope as a function of time, and total lactic acid production. In fact, the production of new AuNPs is rather constant in time and presents a similar slope in the PLAL employing *ω* or *ω *+ 2*ω* pulses, while the slope is progressively reduced during time in PLAL employing green laser pulses, indicating that fragmentation becomes predominant in comparison to the production of new nanoparticles. Finally, the total conversion efficiency into carboxylic acids (*C*_1_ + *C*_2_ + *C*_3_) does not vary significantly under the different experimental LSPC configurations, except for the fragmentation process, with a maximum value of about 10% (Table S3, Supporting Information).

**Figure 3:**
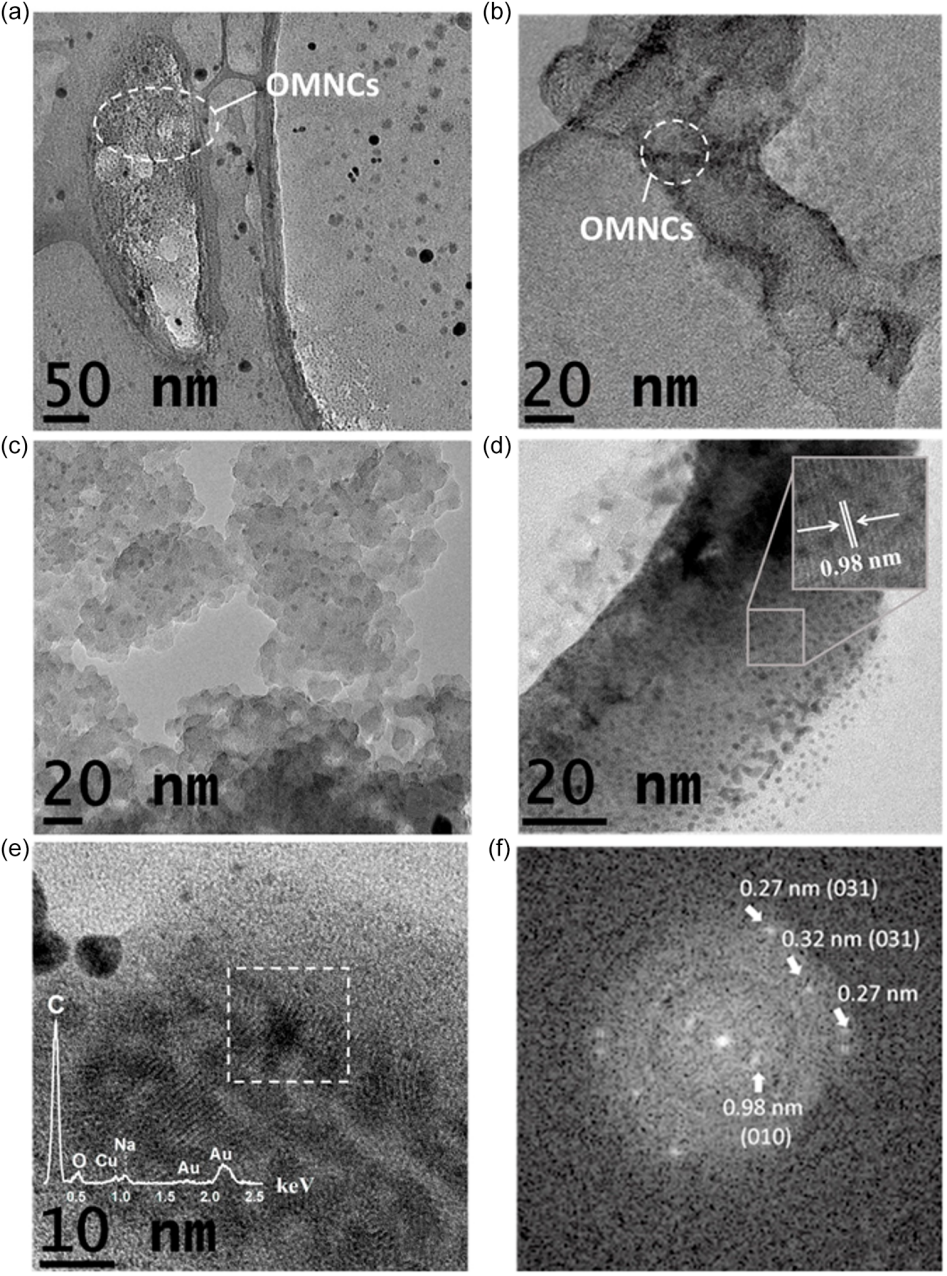
TEM images at an 80 kV acceleration voltage displaying different organometallic nanocomposites (OMNCs) synthesized by the pulsed‐laser‐driven CO_2_RR in water at equilibrium with air, at different *C*_NaOH_ values and different laser pulse configurations. a) AuNCs surrounded by volatile organic material (*C*_NaOH_ = 4.0 mmol L^−1^, *ω*). b) OMNCs composed of AuNCs and amorphous organic material (*C*_NaOH_ = 2.0 mmol L^−1^, 2*ω*). c) Nonvolatile OMNCs synthesized by fragmentation (*C*_NaOH_ = 4.0 mmol L^−1^, *ω* → 2*ω*). d) High‐resolution transmission electron microscopy (HRTEM) image of crystalline OMNCs, demonstrating an interplanar spacing of 0.98 nm (*C*_NaOH_ = 4.0 mmol L^−1^, *ω* + 2*ω*). e) HRTEM image clearly showing OMNCs composed of AuNPs and sodium acetate nanocrystals (*C*_NaOH_ = 4.0 mmol L^−1^, *ω* + 2*ω*), as confirmed by f) the Fourier transforms of the region highlighted by the dashed white square.

Pristine AuNPs colloidal dispersions were further investigated by high‐resolution TEM (HRTEM). **Figure** **3** presents the HRTEM images of different types of organometallic nanocomposites (OMNCs) observed in fresh samples synthesized at different *c*_NaOH_ values and under different laser pulse configurations. Figure 3a highlights a region of the same sample represented in Figure 1b, where small AuNCs of about 2 nm are embedded in the organic material, which volatilizes as soon as a higher magnification (higher electron current density) is applied. A first kind of OMNC stable to electronic irradiation is represented in Figure 3b, where the organic material is characterized by a high degree of disorder and surrounds AuNCs with average dimensions of about 4 nm. Figure 3c shows nonvolatile OMNCs synthesized by fragmentation, where the AuNCs exhibit an average dimension of about 2 nm. A crystalline organic structure supported by AuNPs is represented in Figure 3d, where an interline spacing with an average value of 0.98 nm is observed. Similar OMNCs are shown in Figure 3e, where the elemental energy‐dispersive X‐Ray spectroscopy (EDX) analysis reveals the presence of carbon, oxygen, and sodium in addition to gold. Interestingly, the Fourier transform of the crystal structure (Figure 3f) reveals characteristic sodium acetate interplanar spacings (see Table S4, Supporting Information), demonstrating that the OMNCs depicted in Figure 3d,e are obtained by PLAL‐induced CO_2_RR and *C*_2_ coupling hydrogenation. Most crystalline OMNCs, rarely observed in samples, are extremely sensitive to the acceleration electron voltage applied during the measurements. In fact, as reported in Figure S6, Supporting Information, when the energy is maintained at 200 keV, the interplanar distance associated with the plane (001 sodium acetate) decreases overtime, and the crystal structure rapidly disappears after a few seconds of irradiation.

**Figure 4:**
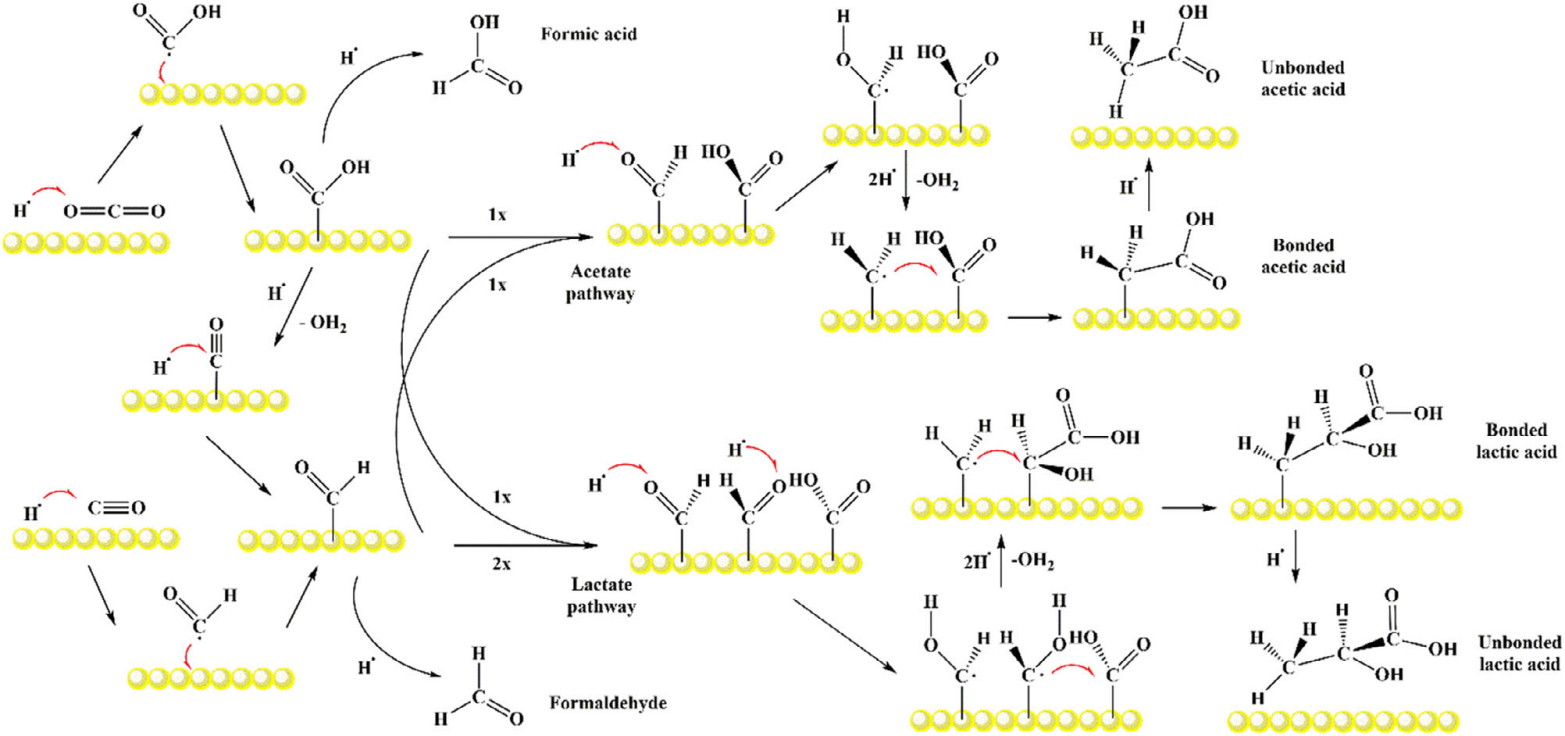
Chemical reactions leading to *C*_2_ and *C*_3_ coupling on gold nanoparticles, producing formic, acetic, and lactic acids in a basic water environment.

The experimental SERS, IC, XRD, and HRTEM results provide clear evidence of CO_2_RR induction by pulsed laser pulses. A detailed display of the chemical reactions leading to the *C*_2_ and *C*_3_ coupling is suggested in **Figure** **4**, for formic, acetic, and lactic acids. As mentioned earlier, PLAL promotes the homolytic cleavage of O—H bond of the water molecule into highly reactive hydroxyl (HO·) and hydrogen (H·) radicals.^[^ ^55^ ^]^ These, in turn, can reduce CO_2_ to CO in aqueous media^[^ ^26^ ^]^ (homogeneous reaction) or, as proposed herein, on the surface of nanoparticles (heterogeneous reaction). In particular, it is proposed that the pulsed‐laser‐driven CO_2_RR to *C*_2_ and *C*_3_ products on gold involves the following three main steps: 1) the reaction of CO_2_ and CO with hydrogen radicals (H**·**), leading to HCO_2_ **·** (carboxyl) and HCO**·** (formyl) radicals, respectively; 2) the binding of HCO_2_ **·** and HCO**·** to nanoparticle surfaces, followed by electron and proton transfers by reaction with hydrogen radicals (H**·**), cleaving C—O bonds and forming C—H bonds (hydrocarbons); and 3) desorption of the final organic products from the nanoparticle surface and diffusion into the bulk of the solution. Indeed, as proposed in ref. [56], acetic acid and lactic acid are produced by the coupled desorption of —CH_3_:—COOH and —CH_3_:—COOH:—CHOH, respectively.

The main proposed acetate and lactate formation pathways on AuNPs start with the reduction of CO_2_ and CO by H**·**, resulting in HCO_2_ **·** and HCO**·** radicals, respectively. The ratio of these radicals on the AuNP can determine the obtained products. As noted in Figure 4, one carboxyl and one formyl molecule are required to obtain an acetate molecule. Furthermore, two formyl and one carboxyl molecules must be close enough to the gold surface to produce lactate. In addition, isolated formyl and carboxyl molecules bound to the gold surface may react with hydrogen radicals to produce formaldehyde and formic acid, respectively. Additionally, methane (CH_4_) may also be obtained by the reaction of formyl with hydrogen radicals (H·), releasing water (H_2_O). Finally, the alkaline medium neutralizes acids due to the presence of sodium hydroxide, yielding sodium acetate and sodium lactate both bound and unbound to AuNPs surface. The fact that formaldehyde was not detected may be associated to its high volatility, resulting in unfavorable conversion or carbonization during the laser ablation process. An alternative *C*_1_ molecule that may be formed comprises methanol, which was also not detected. The steps of the reactions summarizing the proposed mechanisms for obtaining formic, acetic, and lactic acid by the pulsed‐laser‐driven CO_2_RR on gold are reported in Supporting Information.

### 2.3 Photoluminescent OMNCs Characterization

As reported previously, gold PLAL or LFL in water containing NaOH leads to the formation of transparent photoluminescent non‐atomically precise AuNCs.^[^ ^36^, ^57^ ^]^ To characterize their optical emission, the pristine colloidal dispersion containing the nanomaterial was bleached by centrifugation, resulting in a clear dispersion with a gold concentration on the order of 25 ppb. Two distinct emission bands, one in the ultraviolet (UV) and the other in the visible (blue) region, were detected.^[^ ^36^, ^57^ ^]^

**Figure 5:**
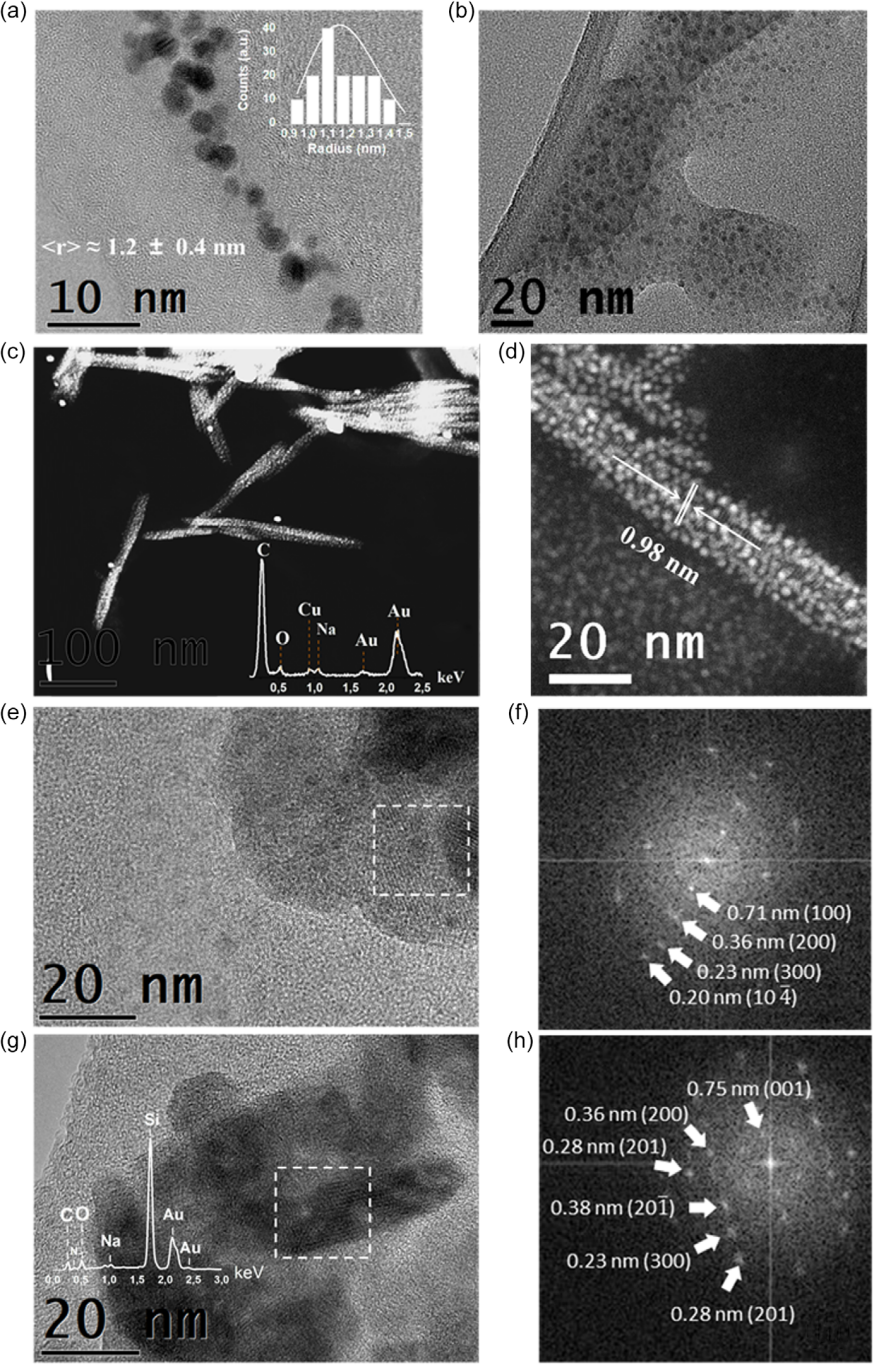
Images of the bleached nanomaterials obtained by HRTEM or HRSTEM with a low electron acceleration voltage (≤100 kV). The nanomaterial was synthesized by PLAL or LFL in water at equilibrium with air at different *C*_NaOH_ values. a) Bare AuNCs (*C*_NaOH_ = 2.0 mmol L^−1^, 2*ω*). b) OMNCs composed of AuNCs and amorphous organic material obtained by fragmentation (*C*_NaOH_ = 4.0 mmol L^−1^, *ω*→2*ω*). The acceleration voltage was set at 100 kV. c,d) HRSTEM images using the high‐angle annular dark‐field detector (HAADF) of OMNCs composed of AuNCs and sodium acetate nanocrystals obtained after filtering the bleached nanomaterial through 0.4 μm filters (*C*_NaOH_ = 4.0 mmol L^−1^, *ω* + 2*ω*). The inset depicts the EDX spectrum of the sample. The interplanar distance corresponding to the plane (0 1 0) plane of sodium acetate is highlighted in (d). e) OMNCs composed of AuNCs and dihydrated sodium formate nanocrystals obtained after filtering the bleached nanomaterial through 0.4 μm filters (*C*_NaOH_ = 4.0 mmol L^−1^, *ω* + 2*ω*). f) The Fourier transforms of the region highlighted by the dashed white square in panel (e). g) The same type of organometallic nanocomposites is displayed in panel (e) but prepared under different synthesis conditions (*C*_NaOH_ = 4.0 mmol L^−1^, 2*ω*). The nitrogen signal noted in the EDX spectra is due to the silicon nitride TEM grid. h) Fourier transform of the region highlighted by the dashed white square in panel (g).

To obtain deeper insights into the effect of the laser‐driven CO_2_RR on morphological and optical AuNCs properties, a bleaching process was applied to the colloidal nanomaterial dispersions synthesized herein. Images of the bleached nanomaterial were obtained by HRTEM or high‐resolution scanning TEM (HRSTEM) at low acceleration voltages, as depicted in **Figure** **5**. Independently of the experimental synthesis parameters, two different types of AuNCs were detected in the samples: the first fully inorganic small ones (diameter between 2 and 3 nm), presented in Figure 5a, and the second, organometallic colloidal nanocomposites composed of AuNCs and organic material in amorphous or nanocrystalline form (Figure 5b–g). When performing LFL, no crystalline organic material was obtained, but rather groups of AuNCs embedded in an amorphous organic matrix, as depicted in Figure 5b. In this case, TEM measurements were not possible at higher magnifications, as this material is highly sensitive to electron irradiation. Some nonvolatile organic crystal structures can instead be observed in PLAL‐obtained samples. In addition to the AuNC–sodium acetate nanocomposites (Figure 5c,d), large colloidal structures composed by AuNCs and dihydrated sodium formate are also clearly visible (Figure 5e,g), as deduced from the interplanar spacings characteristic of the crystals reported in Table S4, Supporting Information, consistent with the Fourier transforms reported in Figure 5f,h. Although these are examples of crystalline organic acids, it is important to note that most organic CO_2_RR products are present in the amorphous phase, as depicted in Figure S7a,b, Supporting Information. The sodium acetate nanocrystals appear both in irregular shapes and in the form of elongated structures, with average transverse and longitudinal dimensions of about 20 and 200 nm, respectively, Figure S7c,d, Supporting Information. The average dimensions of the dihydrated formate nanocrystals are difficult to accurately estimate, due to the difficulty in obtaining dried samples with a low degree of agglomeration. Nevertheless, Figure 5e,g and S7e,f, Supporting Information, suggest that most nanocrystals seem to also exhibit a preferential growth direction, with average dimensions of the order of 50 and 100 nm in the orthogonal direction.

Based on this information, we cannot exclude the possibility that the observed organic structures crystallized during colloid drying over the TEM grid, although the dynamic light scattering results concerning the pristine colloidal solutions reported in Figure S8, Supporting Information, confirm the presence of a bimodal hydrodynamic size distribution, with an average dimension of about 2 and 100 nm, respectively.

**Figure 6:**
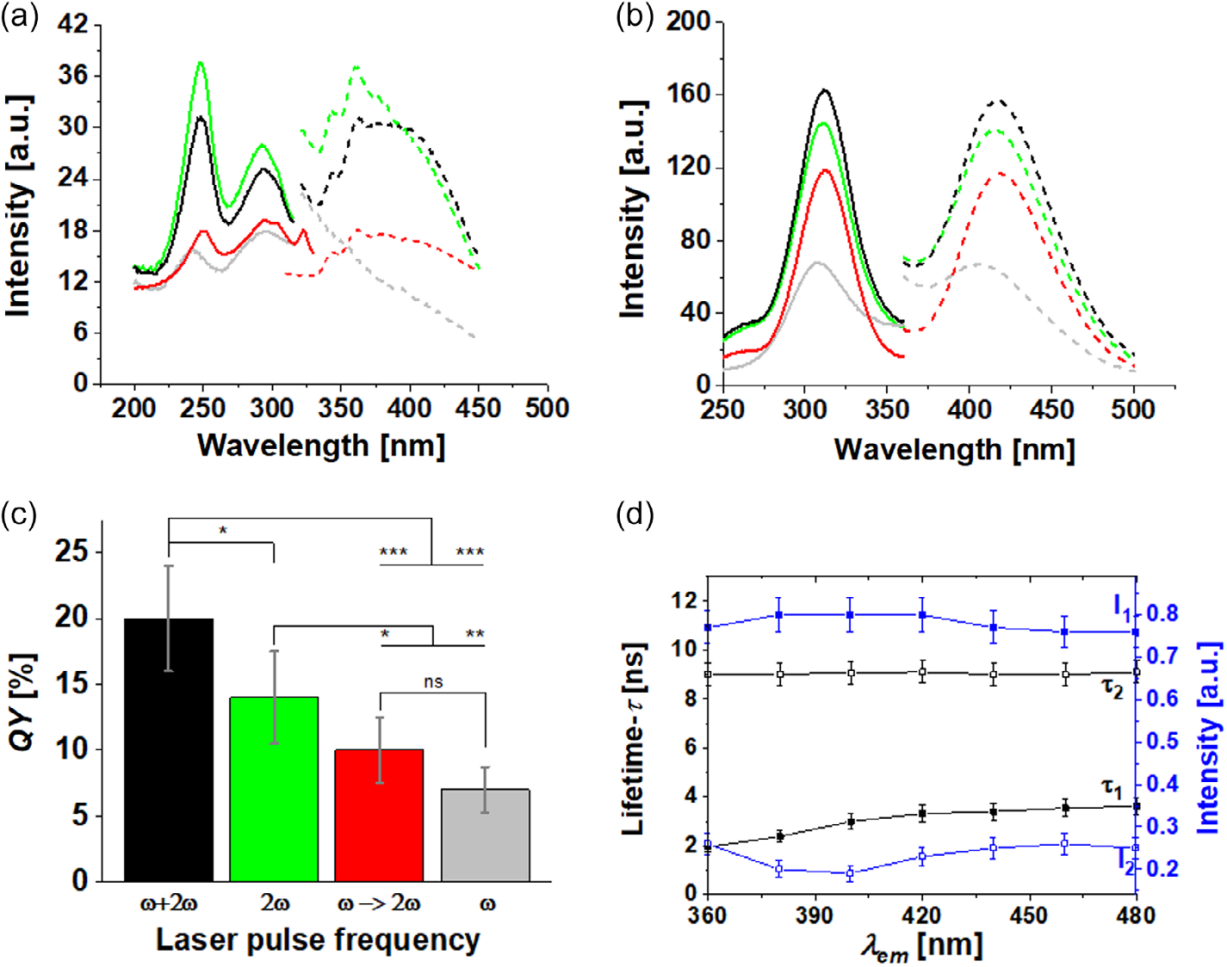
Excitation (solid lines) and emission spectra (dashed lines) of the photoluminescent OMNCs obtained after the bleaching process by centrifugation. The LSPC was performed at the water–air interface with *C*_NaOH_ = 4.0 mmol L^−1^, by LFL (red lines) and PLAL using 2*ω* pulses (green lines), *ω* pulses (grey lines), or *ω* + 2*ω* laser pulses (black lines). The photoluminescence emission in a) the UV and b) visible spectral windows was excited at 250 and 315 nm, respectively. c) *QY* of the photoluminescent nanomaterial with excitation at 304 nm. The vertical lines in the columns represent the standard deviation associated to the measurements of five independent samples. d) Spectral‐dependent *τ*_1_ and *τ*_2_ lifetimes obtained by time‐resolved photoluminescence spectroscopy for the photoluminescent nanomaterials synthesized with *ω *+ 2*ω* laser pulses. The photoluminescence was excited at 304 nm. The data are presented as the means ±  SD. *n* = 5 in panel (c) and *n* = 2 in panel (d). (ns, not significant *p < 0.5, **p < 0.1, and ***p < 0.01).

The optical properties of the nanomaterial obtained after the bleaching process were investigated by steady‐state and time‐resolved photoluminescence spectroscopy. An intense band at about 420 nm (blue band) is present when the material is excited at 315 nm, while a UV band centered at about 360 nm appears when the excitation wavelength is reduced to 250 nm, as shown in **Figure** **6**a,b. The photoluminescence excitation and emission spectra of the OMNCs do not suffer any spectral changes upon laser pulse frequency variations during PLAL, or when LFL is performed. A quantitative optical emission assessment was performed for the samples synthesized under the three different configurations, and the QY was estimated by comparison with a certified QY standard solution (Rhodamine 101) as a function of the sample concentration (e.g., sample dilution). To obtain the number of emitted photons, the optical band was integrated between 315 and 550 nm for the AuNCs and between 540 and 700 nm for Rhodamine 101 (Figure S9, Supporting Information). The comparison of the fairly perfect linear behaviors of the integrated photoluminescence band as a function of absorption (i.e., sample dilution) with the standard (displayed in Figure S10b, Supporting Information) allowed for robust QY assessments. The results presented in Figure 6c demonstrate that the QY can be controlled by the experimental configuration employed in the LSPC. In fact, a value as high as 20% can be obtained by PLAL applying simultaneous *ω* and 2*ω* laser pulses compared to an average value of about 14% and 7% for PLAL applying 2*ω* or *ω* laser pulses, respectively. In addition, the OMNCs synthesized by LFL are characterized by a QY as high as 10%, significantly higher than the QY of 2% recently measured by Ziefuss et al.^[^ ^36^ ^]^ for fully inorganic AuNCs synthesized by LFL. The lowest QY associated to the OMNCs synthesized by PLAL‐driven CO_2_RR with infrared laser pulses was, however, expected. In fact, without the fragmentation process, only a small percentage of primary AuNPs are in the form of small nanoclusters, so the probability of interaction with the produced organic material is low.

Finally, Figure 6d indicates the photoluminescence lifetime of the sample synthesized by simultaneous PLAL with *ω* and 2*ω* pulses for emissions in different spectral regions and excitation at 304 nm. Details concerning the protocols and mathematical approach used to measure the QY and *τ* photoluminescence lifetimes are reported in Supporting Information (Figure S9–S11, Supporting Information).

The photoluminescence decay curves can be properly fitted by applying two different exponential decay curves with *τ*_1_ and *τ*_2_ lifetimes, with their corresponding values given in Table S5, Supporting Information. Traditionally, time‐resolved photoluminescence emission can be used to investigate electron recombination dynamics responsible for the photon emissions that take place in the system. Our samples exhibit a non‐single exponential photoluminescent decay, commonly associated to the presence of different relaxation processes, responsible for different photoluminescence contributions.^[^ ^54^ ^]^ This indicates that different emitting conditions coexist in our samples, as commonly observed when dealing with nanoparticles, due to size and shape distributions that affect their optical properties. Additionally, two different emissive species were detected in our samples, namely small fully inorganic AuNCs and organometallic colloidal nanocomposites composed of AuNCs and carboxylic salts. These conditions justify the occurrence of different photoluminescence decay components. Figure 6d demonstrates that *τ*_2_ is almost constant, with an average value of about 8 ns (<*I*_2_> ≈22%) over the entire emission spectral range, while *τ*_1_ increases from about 1.4–3.0 ns between 360 and 480 nm (≈78%). The lowest experimental *τ*_1_ value is in agreement with the results reported by Ziefuss et al.,^[^ ^36^ ^]^ where the highest lifetime was in the order of 2 ns and was attributed by density functional theory (DFT) calculations to the sp–sp intraband highest occupied molecular orbital‐lowest unoccupied molecular orbital (HOMO–LUMO) transitions of bare AuNCs with dimensions between 2 and 3 nm,^[^ ^32^, ^55^ ^]^ similar to those displayed in Figure 5a. The spectral dispersion observed for *τ*_1_ is consistent with a jellium model approximation, where the measured spectral lifetime may be divided by the intensity of the emission at the respective wavelength to obtain the intrinsic lifetime. The latter, as predicted by spontaneous emission, should increase with decreasing energy emissions.^[^ ^60^ ^]^ The notable differences in comparison with the results reported for the pulsed laser synthesis of fully inorganic AuNCs comprise higher QY and *τ*_2_ values. Regarding the latter parameter, decays in the 5–10 ns range are orders of magnitude below the typical values of AuNCs stabilized by glutathione or thiolate groups,^[^ ^37^, ^61^ ^]^ and are, instead, typical for steric cluster stabilization and spatial confinement by polymers, dendrimers or macromolecules such as vitamin B_1_ or bovine serum albumin.^[^ ^41^, ^60^, ^62^, ^63^ ^]^ The low *τ*_2_ value leads us to conclude that the carboxylic salt crystals located around the nanoclusters do not participate in the ligand to metal charge‐transfer process,^[^ ^37^ ^]^ but rather promote QY enhancement by direct electron donation to the metallic core from the electron‐rich oxygen, as suggested for other sterically stabilized nanoclusters.^[^ ^41^, ^62^ ^]^ Interestingly, carboxylic acids have been reported to play a fundamental role in the formation of colloidal 2D or 3D suprastructures in aqueous media by hydrogen and/or ionic (counter ion) interaction, ultimately leading to the formation of photoluminescent metal‐organic nanoframeworks.^[^ ^64^, ^65^ ^]^ In these particular structures, photoluminescence enhancement and longer lifetimes are due to the inhibition of the degree of freedom of the carboxylic ligands forming the network of metal‐organic nanoframeworks,^[^ ^64^ ^]^ similar to what takes place when the metal‐organic shell is rigidified in ligand‐stabilized luminescent AuNCs.^[^ ^66^ ^]^ Surprisingly, some of the OMNCs in this study present a crystalline organic structure (Figure 5b–h), which should further reduce ligand mobility.

It is important to note that the presence of the organic material produced by the CO_2_RR does not seem to significantly alter the spectral UV and visible (blue) photoluminescence properties, centered around 360 and 420 nm, respectively (Figure 6a,b). In fact, as reported in refs. [36, 57], both fully inorganic AuNCs or AuNCs synthesized by PLAL in water containing low NaOH concentrations (*c*_NaOH_ = 0.02 mmol L^−1^) are characterized by similar UV and visible emission bands, and AuNCs with average dimensions of 2.4 nm exhibit an emission centered around 415 nm. This observation suggests that OMNCs emission is still associated with the HOMO–LUMO transition of metallic cores.

### 2.4 AuNPs Biocompatibility

Since the results presented earlier break the classical paradigm concerning the absence of by‐products during gold PLAL in water, traditionally associated with their high biocompatibility level,^[^ ^67^ ^]^ it seems legitimate to question the viability of the nanomaterial synthesized by the pulsed‐laser‐driven CO_2_RR in a basic water environment. As in vitro toxicity tests are considered the gold standard to evaluate nanoparticle safety, we performed the trypan blue assay on different normal cell lines, namely human keratinocytes (NCTC 2544), murine macrophages (RAW 267.4), and human microvascular endothelial cells (HMVEC) to complete our experimental investigation. A treatment for 24 h with AuNPs synthesized by *ω* + 2*ω* laser pulses at a final concentration of 15 ppm revealed that these nanoparticles were nontoxic and actively taken up in by all tested cells, as displayed in Figure S12, Supporting Information. Thus, the inorganic and organic products obtained during the CO_2_RR do not significantly alter AuNPs biocompatibility, even when the products become macroscopically observable increasing the amount of CO_2aq_ in the ablation environment.

### 2.5 Discussion on the Nature and Effects of the Pulsed‐Laser‐Driven CO_2_RR

**Figure 7:**
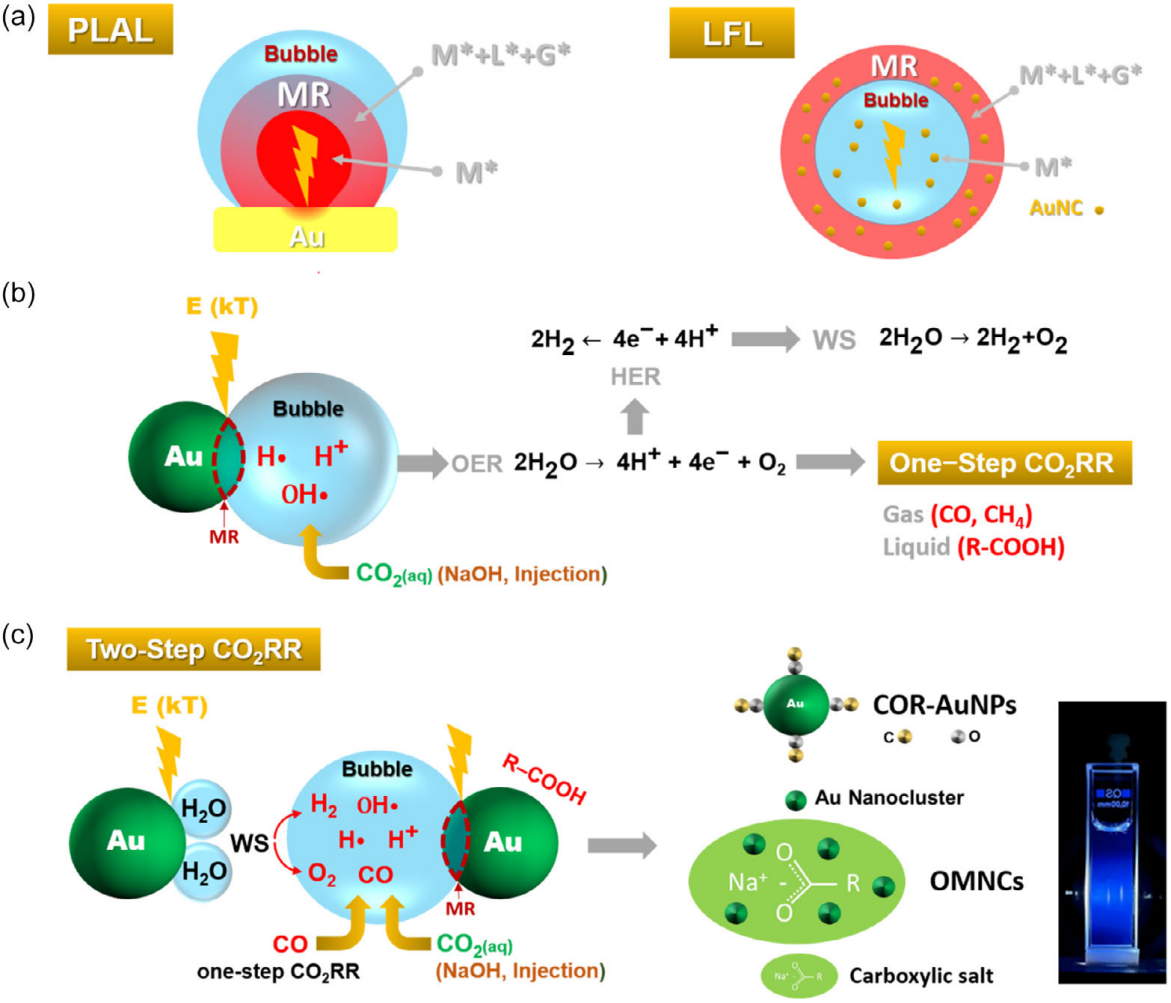
a) Proposed scheme of the physicochemical processes involved in the pulsed‐laser‐driven CO_2_RR with gold during PLAL (left panel) or LFL (right panel). The reaction chamber is formed by the mixing region (MR) at the interface with the laser induced bubble created after laser pulse absorption (orange flash) by the gold target or pristine AuNPs, which supplies the required thermal energy *E*(kT). b) Water splitting (WS) and one‐step CO_2_RR compete with each other, leading to gas (H_2_, O_2_, CO) and possible carboxylic acid (R‐COOH) formation. In the case of PLAL, H• and OH• radicals are also produced during the plasma formation. c) The MR–bubble interface for the two‐step CO_2_RR contains also H_2_ and CO obtained from WS and the one‐step reduction process. CO_2_ can be introduced into the system either by the addition of NaOH or by direct injection. The pulsed‐laser‐driven CO_2_RR process leads to the synthesis of carbon‐monoxide‐rich gold nanoparticles (COR‐AuNPs), and organometallic colloidal nanocomposites composed of AuNCs and carboxylate salts. The photo indicates the visible (blue) photoluminescence of the OMNCs, characterized by a QY of about 20%.

We attempted to provide a phenomenological scenario of the pulsed‐laser‐driven CO_2_RR on gold observed in this study. The temporal evolution of the thermodynamic properties (i.e., temperature and pressure) of the metal–water interface from the laser pulse absorption to final product stabilization has been studied through molecular dynamics simulations by Zhigilei et al. for both the PLAL and LFL processes.^[^ ^18^, ^42^ ^]^ In both cases, a mixing region (MR) is created after a few hundred of picoseconds with an extension of a few tens of nanometers, with temperatures ranging from 10^3^ to 10^4^ K, where excited metal species (M*, liquid droplets and vapors), liquid species (L*, liquid droplets and vapors), and gaseous species (G*) can coexist, and eventually participate in chemical reactions in an environment under a pressure of the order of 10–10^2 ^MPa. In both cases, the MR is created by the interaction between the outer water layer and the phase metal explosion products. The MR depicted in **Figure** **7**a is located at the interface with a cavitation bubble in the expansion phase for PLAL and in the last picosecond before the collapse for LFL.^[^ ^14^, ^42^ ^]^ In the latter case, the MR is above the bubble boundary due to the ejection of AuNCs from the center of the fragmented pristine nanoparticle.^[^ ^14^ ^]^

Our hypothesis is that the CO_2_RR is driven by the thermal energy (*kT*) generated after the laser pulse absorption, shown as flashes in Figures 7b,c, by the target (PLAL) or the AuNPs (LFL). We define the process in which CO_2_ hydrogenation occurs at the surface of the AuNPs (or nanoclusters) by H· radicals or H^+^ ions present in the bubble as a one‐step CO_2_RR. Figure 7b indicates that the electrons resulting from the oxygen evolution reaction can evolve through the hydrogen evolution reaction, which leads to the production of molecular H_2_ by WS, or through the one‐step CO_2_RR, which classically leads to the production of carbon monoxide (CO) and carboxylic acids (R‐COOH). Figure 7c shows the two‐step CO_2_RR, where the bubble may contain H· radicals or H^+^ ions alongside H_2_ produced by WS and CO produced by the one‐step CO_2_RR. The pulsed‐laser‐driven CO_2_RR mechanism is, therefore, probably an overlap of a one‐step and two‐step CO_2_RR, which has recently been proposed as a cascade process to improve the Faradaic efficiency in the synthesis of *C*_2_ and *C*_3_ products for industrial processes.^[^ ^68^ ^]^ As schematically depicted in Figure 7c, the experimental results demonstrate that the pulsed‐laser‐driven CO_2_RR process on gold target leads to the synthesis of various metal‐organic nanostructures, such as carbon monoxide‐rich AuNPs (COR‐AuNPs) and photoluminescent organometallic colloidal nanocomposites composed of AuNCs and carboxylic acids.

The main difference between PLAL and LFL is the absence of a *C*_3_ coupling in the latter process, at least for the laser pulse fluence value applied herein (≈2.5 J cm^−2^). We attribute this difference to the creation of a plasma plume during PLAL which, unlike LFL, is responsible for the presence of strong reducing agents such as H· and H_2_ produced during water‐splitting, and higher transient MR temperatures.^[^ ^19^, ^24^ ^]^ Considering the low values of both the metal concentration and laser pulse fluence employed in the present research, the probability of obtaining molecular hydrogen during LFL by cascade ionization is low.^[^ ^23^, ^24^ ^]^ In this scenario, we propose that both one‐step and two‐step CO_2_RR with molecular hydrogen are involved in the PLAL, while a one‐step CO_2_RR through H^+^ ions is likely to comprise the main conversion pathway during LFL.

We emphasize that the observation of the *C*_3_ coupling to lactic acid during PLAL is the first demonstration of an artificial (photothermal) synthesis of lactic acid in an environment consisting solely of CO_2_ and water. Lactic acid is, in fact, usually obtained by the introduction of additional substances,^[^ ^69^, ^70^ ^]^ glycerol dehydrogenation,^[^ ^71^, ^72^ ^]^ or carbohydrate fermentation supported by lactic acid bacteria,^[^ ^73^ ^]^ demonstrating the unicity of PLAL‐driven CO_2_RR.

Small differences are observed regarding total free carboxylic acid concentrations, depending on the experimental PLAL parameters, and the highest CO_2_ conversion value of about 10% is obtained through the fragmentation of pre‐synthesized AuNPs. The latter result can be understood by considering the fragmentation fluence threshold and cavitation bubble formation dependence on the dimension of pristine AuNPs.^[^ ^74^, ^75^ ^]^ As indicated in Figure 1a, the AuNPs synthesized in deionized water with infrared laser pulses are the only ones that present a significant fraction with diameters higher than 20 nm, characterized by a higher absorption cross section and energy to be used in the thermal processes accompanying the fragmentation.

At first sight, one might expect a correlation between the total CO_2_ conversion efficiency to organic material and the photoluminescent QY of the OMNCs obtained by different experimental LSPC configurations. Conversely, significant differences are observed concerning QY. The main reason is the fact that the organic material detected by IC consists of carboxylic acids in free state, which do not interact with the AuNCs, and do not participate in QY enhancement. Attempts at quantifying the concentrations of both free and bound carboxylic acids by IC analyses of pristine OMNCs were, however, not successful, and damaged the instrument columns during the measurements.

The experimental results also indicate that the PLAL‐driven CO_2_RR by simultaneous irradiation with pulses at the *ω* and 2*ω* frequencies leads to ultrasmall AuNPs with an average diameter of less than 3 nm (Figure 1) and a higher QY of photoluminescent OMNCs (Figure 6c). Both results may be associated to a higher percentage of organic material stabilizing the AuNCs. The latter is responsible for a size‐quenching effect associated with cluster stabilization by organic material during the fragmentation, which could act as an in situ generated surfactant.^[^ ^76^ ^]^ The second experimental observation is consistent with a scenario in which a higher fraction of the photoluminescent AuNCs is embedded in the carboxylic salts produced during the pulsed‐laser‐driven CO_2_RR. Indeed, the HRTEM analysis of the photoluminescent nanomaterial (Figure 5) demonstrated that only a fraction of the AuNCs is stabilized by carboxylic acids, so that the overall QY is reasonably an average of the values associated with the bare and protected nanoclusters.

As a final remark, we emphasize that the present study was aimed at providing a proof of concept of pulsed‐laser‐driven CO_2_RR, and further optimization of both QY and the CO_2_ conversion efficiency is possible. It is legitimate to believe that the CO_2_RR conversion efficiency strictly depends on both the temperature and the concentration of hydrogen reactive species in the MR, as well as bubbles generated during the laser irradiation, as represented in Figure 7.

The temperature of the plasma plume can be generally controlled by the fluence of the laser pulses independently of the temporal duration, and higher temperatures are expected when the laser pulse can interact with the plasma plume along the whole lifetime, which is of the order of ≈100 ns.^[^ ^77^, ^78^ ^]^ The reactive hydrogen species concentration may be, instead, enhanced by exploiting laser water breakdown, due to both cascade ionization and direct medium ionization by multiphoton absorption.^[^ ^79^ ^]^ If cascade ionization can be triggered by the excited AuNPs electrons when nanosecond laser pulses exhibit a fluence of over ≈40 J cm^−2^,^[^ ^23^ ^]^ direct multiphoton absorption can participate in the production of hydrogen when ultrashort laser pulses are considered (ps and fs).^[^ ^80^, ^81^ ^]^ Considering these complex aspects, we deduce that, although the pulsed‐laser‐driven CO_2_RR may be performed by laser pulses with different temporal durations, it is difficult to understand possible differences observed in terms of CO_2_ conversion efficiency at this stage of research. It is instead, straightforward, to predict that the use of state‐of‐the‐art pulsed laser technologies with repetition rates in the order of MHz^[^ ^82^, ^83^ ^]^ may fix the same amount of CO_2_ in a timeframe several orders of magnitude lower than the characteristic time used in the present study, with new perspectives from an energetics point of view.

Interesting results are also expected from the use of dual‐wavelength ultrashort pulses with controllable delay, where the second pulse can interact with primary NPs produced some hundreds of picosecond after the absorption of the first NP by the target.^[^ ^84^, ^85^ ^]^

Although this study does not currently focus on the energetic issues related to CO_2_ conversion, we hope that it will be the starting point for the development of an alternative negative emission technology based on LSPC, where different experimental parameters can be adequately engineered to achieve a competitive CO_2_ conversion rate to advanced nanomaterials in the colloidal phase.

## 3 Conclusions

We demonstrated herein that the CO_2_RR is an intrinsic feature of the gold LSPC at the water–air interface. Carbonyl ligands are present on the surface of AuNPs even when the LSPC is performed in deionized water, while *C*_2_ and *C*_3_ by‐products become macroscopically observable as long as higher amounts of aqueous CO_2_ are introduced via NaOH, creating a basic water environment. Under these conditions, pulsed‐laser‐driven CO_2_RR allows the production of value‐added chemical feedstocks in the colloidal phase. The process is responsible for the production of nanomaterials in the form of carbon‐monoxide‐rich gold nanoparticles and organometallic photoluminescent colloidal nanocomposites consisting of AuNCs and carboxylic salt nanocrystals. While LFL is selective for *C*_2_ coupling to acetic acid, we were able to modulate *C*_2_ and *C*_3_ coupling efficiencies to formic, acetic, and lactic acid by applying laser pulses at different frequencies to perform pulsed laser ablation, also modulating AuNPs dimensions and the QY of the photoluminescent OMNCs. When ablation is performed by the simultaneous irradiation with infrared and green pulses, the synthesis of ultrasmall gold nanoparticles with an average diameter of less than 3 nm was observed, as well as an unprecedented QY as high as 20% in the photoluminescence of the OMNCs, one order of magnitude higher than the characteristic value of fully inorganic AuNCs. These findings shed light on the physicochemical processes underlying LSPC in water and provide the scientific community with a new technological tool for the conversion of CO_2_ into biocompatible advanced nanomaterials in the colloidal phase.

## 4 Experimental Section

The materials used in the present research and the preparation method for the aqueous NaOH solution are reported in Supporting Information.

4.1

4.1.1

##### Laser Synthesis and Processing of the Colloids

The PLAL was performed using laser pulses with a temporal width of 5.8 ns at 532 nm (2*ω*) and 1064 nm (*ω*), generated by a Nd:YAG source operating at a repetition rate of 10 Hz (Q‐Smart 850, Quantel USA). The pulse profile was represented by a TEM00 Gaussian mode, with effective circular beam waist (radius) of 2.8 and 2.6 mm at the *ω* and 2*ω* frequencies, respectively (details reported in Supporting Information). The laser pulses were focused on the target by a focusing lens with focal lengths of 15.2 and 14.9 cm at the *ω* and 2*ω* frequencies, respectively. A polytetrafluoroethylene beaker was used for the synthesis, the height of the water column above the gold target was kept constant at 6 mm, and the same point of the target was ablated for 6 h, for a total number of laser pulses equal to 2.16 × 10^5^. The PLAL was performed under three different laser pulse configurations, namely *ω* pulses, 2*ω* pulses, and simultaneous pulses at both the *ω* and 2*ω* frequencies (*ω *+ 2*ω*). In all three cases (*ω*, 2*ω*, and *ω *+ 2*ω*), the fluence *F* and the energy *E* were calibrated to reach a maximum optical density nanomaterial value after 6 h of PLAL equal to 1.0 ± 0.1. LFL (*ω* → 2*ω*) of preformed AuNPs was performed for 6 h with a laser pulse fluence at the water–air interface of about 2.5 J cm^−2^. Details of the laser pulse energy and fluence in the different experimental configurations for both the PLAL and LFL processes are given in Supporting Information, alongside details concerning the AuNPs synthesis at different water–gas interfaces.

##### Separation of the Transparent Organometallic Photoluminescent Nanocomposites

The photoluminescent OMNCs were obtained from pristine samples by a bleaching process described in detail in ref. [57]. Briefly, the pristine colloidal nanomaterial dispersions were heated at 45 °C under a nitrogen environment, resulting in a tenfold colloidal dispersion concentration, due to mild thermal evaporation. The concentrated samples were then centrifuged at 13 000 rpm and 0 °C for 3 h. Finally, the supernatant was separated from the precipitate, yielding samples containing gold on the order of ≈25 ppb. Transparent nanomaterials exhibiting a high scattering background in the optical extinction spectra were further filtered through 0.4 μm filters.

##### IC, TC, and Metal Concentration Determinations

TC measurements were performed using a total carbon analyzer model TOC–VCPN from Shimadzu (Japan). The experimental setup shown in Figure S3, Supporting Information, was applied to correctly measure the ΔTC (difference in TC of aqueous NaOH solution irradiated with and without the gold target).

The gold concentrations in the water samples were determined by inductively coupled plasma mass spectrometry employing a Nexlon 300X Perkin Elmer apparatus (USA). The samples underwent sonication in an ultrasonic bath for 10 min prior to each measurement.

IC measurements were performed using a DIONEX Thermo‐Fischer ICS‐2000 chromatograph, with automatic eluent generation (KOH), suppressed conductivity detection, and chemical eluent suppression. Details are given in Supporting Information.

##### Optical Spectroscopy Characterization

Sample extinction spectra were obtained using a double beam Lambda 950 Perkin Elmer spectrophotometer (USA). SERS was performed using an XploRA micro‐Raman apparatus (HORIBA), and the samples prepared as explained in Supporting Information. Photoluminescence spectroscopy was performed in both steady‐state and time‐resolved modes, as reported in detail in Supporting Information. The optical QY of the samples was evaluated by comparative method using a Rhodamine 101 reference sample (QY = 100%, details in Supporting Information).^[^ ^86^ ^]^

##### TEM

HRTEM and EDX were performed at different high acceleration voltages (80, 100, 200 kV), depending on sample specificities, as explained in the main text. Two apparatuses were employed throughout the study, a JEOL 2100 F microscope operated at 100 or 200 kV, and a FEI Titan 80‐300 microscope operated at 80 kV. Details are given in Supporting Information.

##### XRD

XRD measurements were performed using a single‐crystal Oxford Diffraction SuperNova Agilent diffractometer (USA) equipped with a Titan CCD detector, using CuK*α* radiation (1.5416 Å) at room temperature. The crystalline phases present in the experimental samples were identified using the Qual‐X suit program for the qualitative and quantitative phase analyses (QPAs).^[^ ^83^ ^]^ The QPA was performed using the TOPAS Academic system^[^ ^84^ ^]^ applying Rietveld method.^[^ ^85^ ^]^ Details are reported in Supporting Information.

##### Statistical Analyses

The LabSpec6 (HORIBA) and OriginLab 2019b software were used to process the SERS data, where the baseline was removed using a seventh‐degree polynomial, and the spectra were shifted along the vertical axes for better visualization. The UV–vis spectra were normalized at a value of 1. The XRD data were refined by the Rietveld method using the DIFFRAC TOPAS‐R software available in the Bruker AXS Version 4.2 and the baseline was removed using OriginLab 2019b with a thirty point interpolation. The data were presented as the means ± standard deviation (±SD) for each group, and “*n*” represents the number of samples per group. A one‐way ANOVA was used for intergroup comparisons when more than two groups were analyzed. ns indicates that there is no statistically significant difference, while *p < 0.5, **p < 0.1, ***p < 0.01, ****p < 0.001.

## Conflict of Interest

The authors declare no conflict of interest.

## Supporting information

Supplementary Material

## Data Availability

The data that support the findings of this study are available from the corresponding author upon reasonable request.
