# Correction to “Pulsed‐Laser‐Driven CO_2_ Reduction Reaction for the Control of the Photoluminescence Quantum Yield of Organometallic Gold Nanocomposites”

**DOI:** 10.1002/smsc.202400569

**Published:** 2024-11-27

**Authors:** Tahir, Guilherme C. Concas, Mariana Gisbert, Marco Cremona, Fernando Lazaro, Marcelo Eduardo H. Maia da Costa, Suellen D. T. De Barros, Ricardo Q. Aucélio, Tatiana Saint Pierre, José Marcus Godoy, Diogo Mendes, Gino Mariotto, Nicola Daldosso, Francesco Enrichi, Alexandre Cuin, Aldebarã F. Ferreira, Walter M. de Azevedo, Geronimo Perez, Celso SantAnna, Braulio Soares Archanjo, Yordy E. Licea Fonseca, Andre L. Rossi, Francis L. Deepak, Rajwali Khan, Quaid Zaman, Sven Reichenberger, Theo Fromme, Giancarlo Margheri, José R. Sabino, Gabriella Fibbi, Mario Del Rosso, Anastasia Chillá, Francesca Margheri, Anna Laurenzana, Tommaso Del Rosso

*Small Sci.* **2024**, *4*, 2300328, https://doi.org/10.1002/smsc.202300328

In section S.1. of the Supporting Information, the text “The resulting liquid was then stirred for a further 24 h in the open air at 4 °C and stored in separate 15 ml Falcon tubes. Each Falcon tube was opened just prior to the PLAL process.” was incorrect. This should have read: “For Pulsed Laser Ablation process, the resulting liquid was further stirred 24 h in the open air at 4 °C and stored in separate 15 ml Falcon tubes. Each Falcon tube was opened just prior to the PLAL process. For the fragmentation process, calibrated aliquots of NaOH were added to the colloidal dispersion of AuNPs produced in deionized water. In this case, the laser irradiation was started immediately after the introduction of NaOH. Once finished the PLAL or fragmentation process, the samples were protected with parafilm and stored at 4 °C.”

In Table S4, Supporting Information of section S.5. Ion chromatography, the text “As reported in Table S2, Supporting Information, the average TC of the samples was 35 ppm.” was incorrect. This should have read: “The average TC of the samples was 35 ppm for PLAL and 20 ppm for the fragmentation process.”

We apologize for these errors.

