# Supplementary material for: Pulsed‐Laser‐Driven CO2 Reduction Reaction for the Control of the Photoluminescence Quantum Yield of Organometallic Gold Nanocomposites
Source: Small Sci. 2024 Apr 1;4(7):2300328. doi: 10.1002/smsc.202300328 (PMC11935044; doi:10.1002/smsc.202300328)
Supplement: Supplementary file 1 — Supplementary Material [file SMSC-4-2300328-s001.pdf]

Supporting Information

Pulsed laser driven CO_2 reduction reaction for the control of the photoluminescence quantum yield of organometallic gold nanocomposites

Tahir, Guilherme C. Concas, Mariana Gisbert, Marco Cremona, Fernando Lazaro, Marcelo Eduardo H. Maia da Costa, Suellen D. T. De Barros, Ricardo Q. Aucélio, Tatiana Saint Pierre, José Marcus Godoy, Diogo Mendes, Gino Mariotto, Nicola Daldosso, Francesco Enrichi, Alexandre Cuin, Aldebarã F. Ferreira, Walter M. de Azevedo, Geronimo Perez, Celso SantAnna, Braulio Soares Archanjo, Yordy E. Licea Fonseca, Andre L. Rossi, Francis L. Deepak, Rajwali Khan, Quaid Zaman, Sven Reichenberger, Theo Fromme, Giancarlo Margheri, José R. Sabino, Gabriella Fibbi, Mario Del Rosso, Anastasia Chillà, Francesca Margheri, Anna Laurenzana and Tommaso Del Rosso

S.1. Materials and preparation of the aqueous NaOH solution

Materials:

Sodium carboxylate salts, sodium hydroxide (NaOH), NaCl, dibasic potassium phosphate, Rhodamine 101, trypan blue and Dulbecco's Modified Eagle Medium (DMEM), were purchased from Sigma-Aldrich. Gold targets with 99% purity were purchased from the Kurt J. Lesker Company (U.S.A). Deionized water had a conductivity equal to 18.2 MΩ.cm and was obtained using a Milli-Q purification system. The wet cellulose membranes for dialysis (3.5 KD molecular weight cut-off, 10% glycerol, 0.1% sulfur) were purchased from Spectrum Laboratories (U.S.A).

Preparation of the aqueous NaOH solution:

A stock solution of sodium hydroxide in deionized water at a concentration of 100 mM was stirred in a closed recipient for 30 minutes. Calibrated aliquots of the stock solution were added to 250 ml of dionized water to obtain the desired NaOH concentration. The resulting liquid was then stirred for a further 24 hours in the open air at 4 ^{o}C, and stored in separate 15 ml Falcon tubes. Each Falcon tube was opened just prior to the PLAL process.

S.2. Experimental setup for the LSPC

Figure S1 shows the experimental set-up used for PLAL with laser pulses at different frequencies: ω (1064 nm), 2ω (532 nm), and with simultaneous pulses at 1064 nm and 532 nm (ω + 2ω). The Nd:YAG laser source is a model Q-Smart 850 (Quantel U.S.A, repetition rate of 10 Hz) with a pulse duration τ_{laser} = 5.8 ns. For the simultaneous PLAL with pulses at

both ω and 2ω frequencies, the infrared beam dumper (ω pulses) is eliminated from the head of the 2ω-frequency module of the laser source. As shown in Figure S1, in the front of the exit of the ω pulses are located a series neutral density filters (NDFs) and a dichroic mirror (DM_1) transparent for ω pulses and reflecting 2ω pulses (model DMLP900, ThorLabs U.S.A). Another dichroic mirror (DM_2) is placed at the exit of the 2ω pulses on the head of the laser, and used together with DM_1 to overlap the spot of the ω and 2ω pulses. The overlap is checked by two pinholes located in the optical path common to the pulses of different frequency, used only in the alignment step and not visible in the drawing. The pulses are then deflected perpendicularly by a mirror (not shown in the Figure for simplicity) and focused on the target by a focusing lens L with focal lengths of 15.2 and 14.9 cm at ω and 2ω frequencies, respectively. PLAL was performed in a becker of polytetrafluoroethylene (PTFE), and the height and volume of the water column over the gold target were kept fixed at 6 mm and 8 ml, respectively.

To calculate the diameter and fluence F of the laser pulse on the target, all the above parameters were taken as input to a code based on the ABCD matrix method [1], which simulates the propagation of circular Gaussian beams passing through focusing lenses, glass optical elements and water. Considering the manufacturer specifications, the laser spot of the Nd:YAG source used in the experiments is characterized by a TEM00 Gaussian profile, and is elliptical with minor differences between the major and minor axis (less than 5%). For this reason, the laser spot has been considered circular in the optical simulations, with effective values of the beam waist (radius) of 2.8 mm and 2.6 mm at the ω and 2ω frequencies, respectively, as verified by angular divergence measurements.

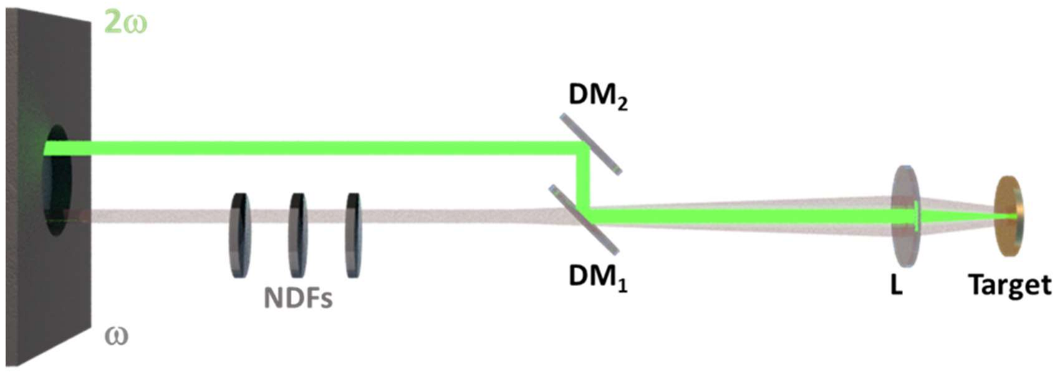

Fig.S1 Experimental setup used for PLAL. The laser source was a model Q-Smart 850 (Quantel U.S.A, repetition rate of 10 Hz) with a pulse duration $\tau_{laser} = 5.8$ ns.

In all the three cases (ω, 2ω, and ω+2ω), the fluence *F* and energy *E* were calibrated in order to reach a maximum value of the optical density of the nanomaterial after 6 hours of PLAL equal to (1.0 ± 0.1). Table S1 shows the laser pulse parameters for the different configurations: ϕ is the diameter of the spot on the target, *E* is the energy, and *F* is the fluence impinging on the surface of the gold target, without the presence of the AuNPs. The initial total fluence *F* (at time $t = 0$ s) of the laser pulses over the gold target was 3.5 J/cm^2 for PLAL with 2ω pulses, 5.5 J/cm^2 for pulses at ω frequency, and 4.5 J/cm^2 for (ω+2ω) laser pulses. In the latter case, we have expressed the total fluence as $F_{\omega+2\omega} = (F_{\omega} + F_{2\omega})$, and F_{ω} is fixed at a value of about 2.4 J/cm^2, which is near to the ablation threshold value at the wavelength of 1064 nm [2, 3].

To perform LFL, AuNPs were first synthesized by PLAL in deionized without NaOH using ω laser pulses with a fluence F = 8.2 J/cm^2, for a total time of 3 hours. After the synthesis, 4mmol/L of NaOH was added to the colloidal dispersion of the AuNPs, which was diluted in order to obtain a gold concentration of about 80 ppm. Subsequently, the AuNPs were stored in a becker of PTFE, forming a column of liquid with a height of 6 mm, and irradiated with laser pulses at 2ω frequency for a total time of 6 hours (same height of the liquid and same number of laser pulses used to perform PLAL in water with NaOH). Differently from the PLAL process, during LFL the laser pulse was focused about 1 cm above the surface of the liquid, and the optical system was calibrated to obtain a laser pulse fluence at the water-air interface of about 2.5 J/cm^2. A magnetic stirrer was used during LFL.

Laser Pulses	ϕ_{ω} [µm]	$\phi_{2\omega}$ [µm]	E_{ω} [mJ]	$E_{2\omega}$ [mJ]	F_{ω} [J/cm^2]	$F_{2\omega}$ [J/cm^2]	F_{total} [J/cm^2]
ω	400	-	8.0	-	5.5	-	5.5
2ω	-	400	-	5.0	-	3.5	3.5
ω+2ω	560	400	7.0	3.0	2.4	2.1	4.5
ω –> 2ω	400	400	12.0	5.0	8.2	3.5	-

Table S1. Experimental parameters of the laser pulses used to perform LFL (ω→2ω) and PLAL at different frequencies (ω, 2ω, and ω+2ω). In the case of PLAL, the parameters *ϕ*, *E* and *F* denote the diameter, energy and fluence of the laser pulses on the target, respectively. In this case, the values of energy and fluence are calculated in the absence of the AuNPs and therefore represent the initial (t = 0 s) values of the parameters. In the case of LFL, the values of *ϕ*, *E* and *F* denote the diameter, energy and fluence of the laser pulses at the interface between the air and upper surface of the colloidal dispersion of the pre-synthesized AuNPs.

In Figures S2(a-d) are represented the time evolution of both the maximum optical extinction of the colloidal nanomaterial and the total laser pulse fluence F incident on the target, when PLAL is performed using laser pulses at different frequencies. The calculation has been performed considering the absorption of the energy at the 2ω frequency by the pre-formed gold nanoparticles considering a liquid height of 6 mm, and disregarding the shielding effect of the cavitation bubbles [4].

As visible in Figures S2(b,d), the fluence of the laser pulses at the 2ω frequency decreases as the value of the optical extinction increases in time. Therefore, in order to obtain a continuous production of AuNPs, the energy of the pulses was raised at the time indicated by the dashed grey lines in Figures S2(a,c). In particular, $E_{2\omega}$ was increased from 5.0 to 6.0 mJ when performing PLAL with only 2ω pulses, while in the (ω+2ω) configuration, $E_{2\omega}$ was increased from 3.0 to 4.0 mJ, and E_{ω} from 7.0 to 8.0 mJ. There was no temporal variation in pulse energy when PLAL was performed with ω pulses.

The analysis of panels (b) and (d), shows that when 2ω pulses are used, in the last half time of the PLAL process, $F_{2\omega}$ is always between 1.0 and 1.5 J/cm^2, so that the gold nanomaterial is subjected to a simultaneous process of ablation and fragmentation in liquid [5, 6], which does not happen when only infrared laser pulses are used.

In Figure S2(e) is reported the time evolution of the normalized extinction spectra of the AuNPs produced by pure fragmentation. The reduction of the size of the AuNPs during LFL is evident in Figure S2(f), where the extinction in the region of the interband transition of gold at 440 nm, first increases in time from 0.61 to 0.70 and finally stabilize at a value of 0.68 [7], [8].

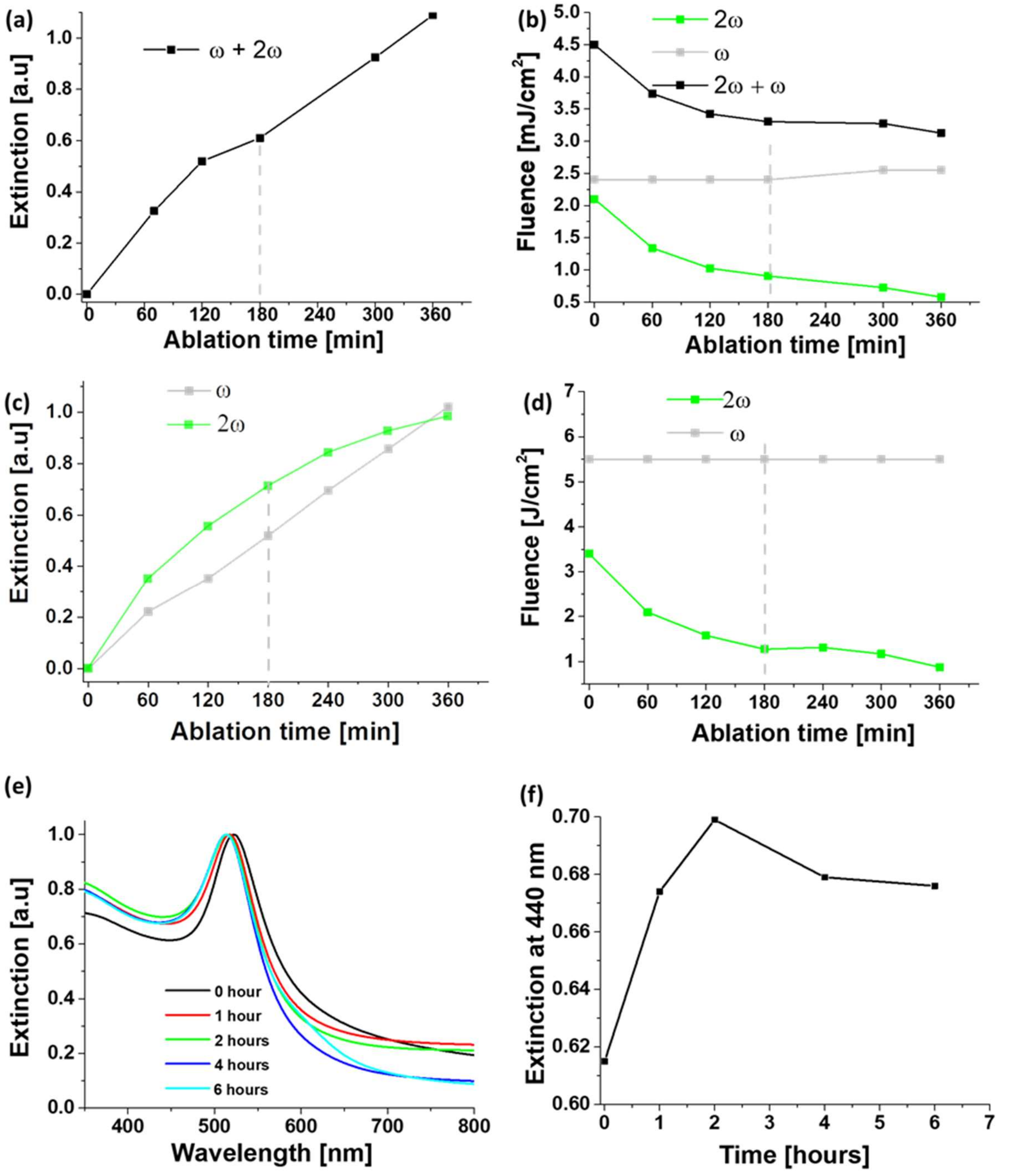

Fig.S2 Temporal dependence of the maximum value of the optical extinction of the colloidal dispersion of the nanomaterial (a,c), and of the fluence of the laser pulses F impinging on the target (b,d) during PLAL. (a,b) PLAL performed by simultaneous pulses at ω and 2ω frequencies. (c,d) PLAL performed with ω or 2ω pulses. The grey dashed lines in all panels indicate the time at which the energy of the 2ω laser pulses was increased. (e) Temporal evolution of the normalized extinction spectra of the AuNPs during LFL. (f) Temporal evolution of the value of the extinction of the AuNPs at 440 nm during LFL.

S.3. Measurement of ΔTC

In the main text, the parameter ΔTC indicates the difference in the total carbon (TC) of the samples obtained by PLAL of the gold target and irradiation of a water solution (without gold target or AuNPs) with the same NaOH concentration (TC_0), so that $TC = (TC_0 + \Delta TC)$. To correctly measure ΔTC we used the experimental setup shown in Figure S3, where the laser pulse is directed in two orthogonal directions using of a 50:50 beam splitter (BS). Two mirrors M_1 and M_2 deflect them to the lenses L_1 and L_2, with the same focal distance. One ablation line is occupied by the NaOH water solution with the gold target, while in the second one we simply put the aqueous NaOH solution, without any target.

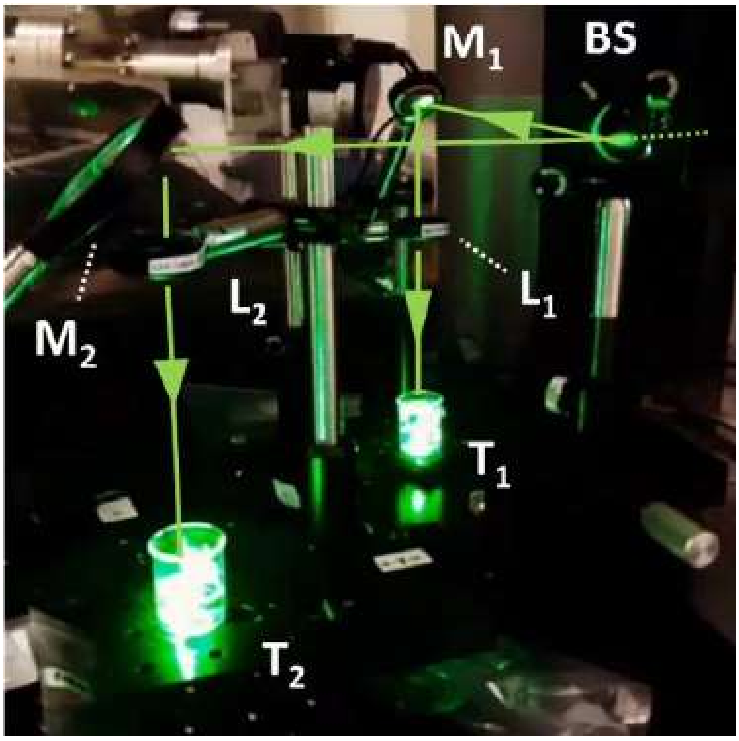

Fig.S3 Experimental setup used for the preparation of the samples used to measure the total carbon variation ΔTC during the PLAL of the gold target.

In Table S2 we report the characterization of the nanomaterial synthesized by PLAL with pulses at different frequencies. C_{Au} represents the concentration of gold in the samples.

Laser Pulses	C_{NaOH} [mmol/L]	*pH* [initial] –> *pH* [final]	TC_0 [ppm]	ΔTC [ppm]	TEM *Radius* [nm]	C_{Au} [ppm]
**2ω**	0.06	9.5 ± 0.2 –> 6.8 ± 0.2	6 ± 2	0.5 ± 0.1	2.3 ± 0.7	72 ± 2
**2ω**	2.00	11.0 ± 0.1 –> 8.1 ± 0.4	22 ± 5	3 ± 1	1.9 ± 0.3	80 ± 4
**2ω**	4.00	11.3 ± 0.1 –> 9.3 ± 0.3	31 ± 6	4 ± 1	1.7 ± 0.8	82 ± 4
**ω+2ω**	2.00	11.0 ± 0.1 –> 8.1 ± 0.4	22 ± 5	3 ± 1	1.6 ± 0.4	81 ± 3
**ω+2ω**	4.00	11.3 ± 0.1 –> 9.3 ± 0.3	31 ± 6	4 ± 1	1.3 ± 0.3	80 ± 5
**ω**	0.00	6.9 ± 0.5	1.0 ± 0.5	0	9.4 ± 4.2	100 ± 6
**ω**	4.00	11.3 ± 0.1 –> 9.3 ± 0.3	31 ± 6	4 ± 1	3.9 ± 2.0	62 ± 2

Table S2. Full analytical and dimensional characterization of the samples obtained by PLAL. The standard deviations in the parameters TC_0 and ΔTC were evaluated considering the results obtained for about 30 independent processes over 3 years for each value of C_{NaOH}.

S.4. SERS

For the SERS investigation, AuNPs were synthesized by PLAL with laser pulses at 2ω frequency at different water-gas interfaces without NaOH: argon ($AuNPs_{AR}$), nitrogen ($AuNPs_{N2}$), and a mixture of 1% CO_2 and 99% argon ($AuNPs_{CO2}$, percentage relative to the partial pressure). To control the gaseous atmosphere in contact with the liquid during PLAL, a vacuum was created in the ablation glass chamber containing the deionized water with the target for 60 s. Later, the desired gas was fluxed inside the chamber and then closed, and the gas mixture was allowed to diffuse into the water at a pressure of about 1.2 Atm for about 5 hours before performing the PLAL process.

Surface Enhanced Raman spectroscopy was performed using a micro-Raman model XploRA (HORIBA), equipped with a CCD detector and a thermoelectric cooling system (operating temperature -50 ºC), at a wavelength of 638 nm. For the preparation of the samples, 6 drops of the pristine material with a volume of 40 μL were deposited on a clean glass as substrate,

each one dried at the temperature of 40°C. The final samples were characterized by a well-defined Localized Surface Plasmon Resonance at the excitation wavelength.

In Figure S4 we report the SERS spectra in the gold-carbonyl region of the nanomaterial synthesized by 2ω pulses in deionized water in equilibrium with different gaseous atmospheres, without and with the addition of 2 mmol/L NaOH after PLAL. For comparison, the Raman spectrum of the lyophilized powders of the nanomaterial is also shown.

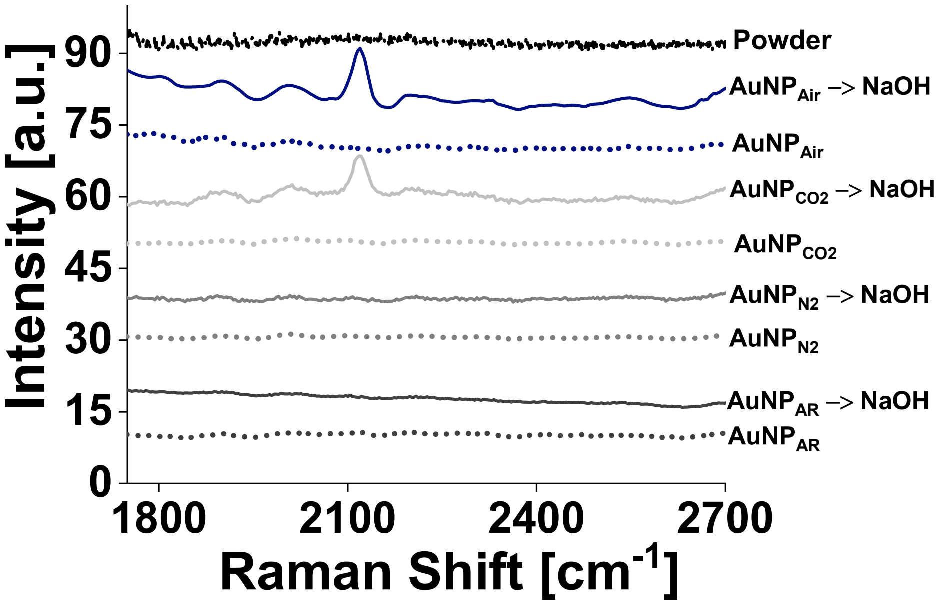

Fig.S4 SERS spectra of nanomaterial synthesized by PLAL in deionized water without NaOH with pulses at 2ω frequency (dotted lines). The water was in equilibrium with argon ($AuNP_{AR}$), N_2, air, or 99% CO_2 with 1% of Argon ($AuNP_{CO2}$). For each case, the SERS spectra of the nanomaterial upon addition of 2 mmol/L NaOH after PLAL are also shown (continuous lines). The dashed spectra correspond to the Raman response of the lyophilized powder of the nanomaterial, synthesized in water with C_{NaOH} = 2.0 mmol/L in equilibrium with air. The SERS spectra were shifted along the vertical axis for a better visualization.

The introduction of 2 mmol/L NaOH after the synthesis (→ NaOH) changes the pH of the colloidal dispersion of the nanomaterial from 6.0 to 8.0. The altered pH gives the proper stability to the colloidal dispersion, so that the nanomaterial does not precipitate during the deposition of the droplets on the silicon substrate used to perform SERS, and creates the proper localized surface plasmon resonance (LSPR) band necessary to obtain an amplified Raman response. This is the reason why SERS signal for $AuNP_{CO2}$ appears after the introduction of the hydroxide.

The Raman spectrum of the lyophilized powder does not show the signal of gold carbonyl, but shows a line around 1060 cm^{-1} (not shown), which is attributed to the trona and nahcolite detected by XRD (Figure 2(c)) in the main text.

S.5. Ion chromatography

Ion Chromatography measurements were performed using a DIONEX Thermo-Fischer ICS-2000 chromatograph, with automatic eluent generation (KOH), suppressed conductivity detection, and chemical suppression of the eluent. The system uses two columns (Dionex IonPac AS18 Analytical 4 × 250 mm, and Dionex IonPac AG18 Guard 4 × 50 mm), and was operated with an elution gradient from 1.50 to 40.0 mmol/L at a flow rate of 1.0 mL/min and back pressure of ~2500 psi, for a total run time of 22 min. The system was calibrated using standard aqueous solutions of carboxylic acids (lactic, acetic, propionic, formic and butyric), prepared gravimetrically with the appropriate sodium-based salt. An initial solution of 1000 mg/L was prepared for each carboxylic acid, followed by the preparation of a standard solution containing the different carboxylic acids at individual concentrations of 10 mg/L. The mixed standard solution was used to construct two calibration curves in the ranges between (1.0 to 10.0) mg/L and (0.10 to 1.0) mg/L.

In Figure S5 are represented the elution time curves relative to the ion chromatography measurements on the fraction of the Au derived nanomaterial diffusing through the pores of a dialysis membrane (3.5 KD). PLAL was performed by laser pulses with different frequencies, in water at equilibrium with air and with $c_{NaOH} = 4.0$ mmol/L. Together with the results obtained with the synthesized samples, the chromatogram related to the calibrated aqueous solution containing the mixture of carboxylic acids at a concentration of 1 ppm is also shown in panel (a). In order to obtain the concentrations of the carboxylic acids, a calibration curve was established using reference samples with concentrations ranging from 1 to 10 ppm. The calibration curves were used to determine the elution time corresponding to each carboxylic acid and to quantify in ppm the concentration of the analytes in the synthesized samples by integrating the signals of the elution curve over time.

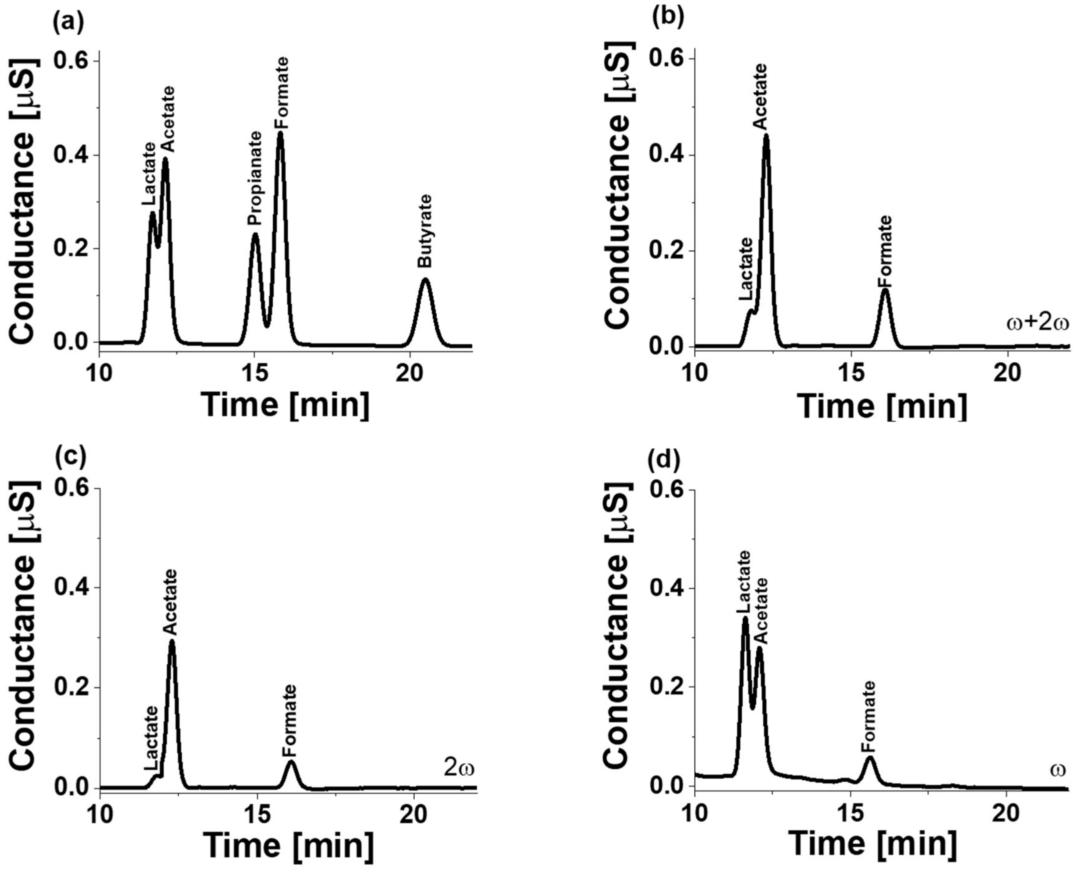

Fig.S5 (a) Chromatogram relative to the standard aqueous solution of mixed carboxylic acids at the concentration of 1 ppm. (b-d) Elution time curve of the ion chromatography performed on the transparent material obtained after the dialysis process with 3.5 kD membrane. PLAL was performed in water containing 4 mmol/L of NaOH in equilibrium with air, with simultaneous pulses at ω and 2ω frequencies (b), with pulses at 2ω frequency (c), and with pulses at ω frequency (d).

In the case of the samples obtained by diffusion through the dialysis membranes (3.5 kD), 20 ml of the fresh colloidal dispersion of the nanomaterial were dialyzed with 200 ml of deionized water in the environment external to the membrane. After 4 days of magnetic stirring, the external liquid was concentrated to the initial volume of the sample (i.e. 20 ml) by heating at 50°C in nitrogen atmosphere, and finally analyzed by ion chromatography.

In Table S4 we report the concentrations of the carboxylic ions detected by ion chromatography on samples produced by LSPC, using water with c_{NaOH} = 4.0 mmol/L in equilibrium with air. The coupling efficiencies are calculated by dividing the concentration of the specific product by the total carbon TC = 35 ppm.

Laser Pulses	C_{NaOH} [mmol/L]	<TC> [ppm]	Processing Method	<Formate> [ppm] Conversion Efficiency (C_1 Coupling) [%]	<Acetate> [ppm] Conversion Efficiency (C_2 Coupling) [%]	<Lactate> [ppm] Conversion Efficiency (C_3 Coupling) [%]	$C_1 + C_2 + C_3$ Conversion Efficiency	C_3/C_2
ω	4.00	35.0 ± 6.0	Dialysis and Heating	0.20 ± 0.05<br>0.6 %	1.0 ± 0.3<br>2.9 %	1.2 ± 0.3<br>3.4 %	6.9 %	1.20
2ω	4.00	35.0 ± 6.0	Dialysis and Heating	0.30 ± 0.08<br>0.8 %	1.4 ± 0.4<br>4.0 %	0.20 ± 0.05<br>0.6%	5.4 %	0.14
ω+2ω	4.00	35.0 ± 6.0	Dialysis and Heating	0.4 ± 0.1<br>1.1 %	1.7 ± 0.4<br>4.8 %	0.5 ± 0.1<br>1.4 %	7.3 %	0.29
ω –> 2ω	0	20.0 ± 5.0	Dialysis and Heating	-	2.0 ± 0.5<br>10.2 %	-	10.2 %	0

Table S4. Concentration of the carboxylic acid ions measured by ion chromatography. The samples were obtained by PLAL or LFL in water with C_{NaOH} = 4.0 mmol/L in equilibrium with air. The analyzed organic material was obtained by diffusion of the pristine colloidal dispersion of the nanomaterial through a 3.5 kD dialysis membrane. As reported in Table S2, the average *TC* of the samples was 35 ppm. The data are presented as the means ± SD (n = 5).

Based on the scheme proposed in Figure 4 of the main text, the reactions leading to sodium acetate and lactate by PL-CO_2RR can be expressed as follows:

1) $CO_2 + 3H\cdot \rightarrow H_2O + HOC\cdot$
2) $HOC\cdot + H\cdot \rightarrow HOCH$ (formaldehyde)
3) $HOC\cdot + 5\,H\cdot \rightarrow H_2O + CH_4$ (methane)
4) $CO_2 + H\cdot \rightarrow HOOC\cdot$
5) $HOOC\cdot + H\cdot \rightarrow HOOCH$ (formic acid)
6) $HOC\cdot + HOOC\cdot + 4H\cdot \rightarrow H_3C_2OOH + H_2O$
7) $H_3C_2OOH + NaOH \rightarrow H_2O + H_3C_2OONa$ (sodium acetate)
8) $2HOC\cdot + HOOC\cdot + 5\,H\cdot \rightarrow H_5OC_3OOH + 2\,H_2O$
9) $H_5OC_3OOH + NaOH \rightarrow H_2O + H_5OC_3OONa$ (sodium lactate)

S.6. X-Ray diffraction

The pristine colloidal dispersions of nanomaterial synthesized by PLAL in water containing different concentrations of sodium hydroxide (0.05 mmol/L, 1.00 mmol/L, 2.00 mmol/L and 4.00 mmol/L) in equilibrium with air, were dried in 2 mL volume microtubes using a vacuum centrifuge from Eppendorf (Germany) model Vacufuge Plus. After drying, the nanomaterial pellets were mounted on nylon loop supports (Hampton Research, USA) and subjected to diffraction analysis. X-ray diffraction measurements were performed using a single crystal

diffractometer from Agilent (U.S.A, model Oxford Diffraction SuperNova) equipped with a Titan CCD detector, using CuKα radiation (1.5416Å) at room temperature. The diffractometer was operated at a voltage of 50 kV and a current of 0.8 mA. Diffraction data were collected by 2θ angular scanning in steps equal to 0.0263º. Data acquisition was performed using the CrysAlisPro software (Oxford Diffraction Ltda). For Quantitative Phase Analysis (QPA), the refinement step was carried out in the range 6.5 to 125 (2nd), and cell parameters of each crystalline phase were refined including 7 parameters for the background, modeled by a Chebyshev polynomial function. Moreover, the atomic positions of the compounds were kept fixed. The R_{wp} and GOF (goodness-on-fit) values were 7.2 and 0.084 and the R_{Bragg} values of Gold, Trona, NaHColite and Sodium Acetate were: 4.49; 5.66; 4.39; and 10.21, respectively.

In Table S3 we report the parameters of the crystal structure of metallic gold, of the carboxylic acids detected by ion chromatography, of trona and nahcolite. The table was used for the interpretation of the XRD and high resolution electron microscopy measurements (HRTEM). The values for trona, nahcolite and sodium acetate are based on the JCPDS database [9] (reference codes). The values for sodium di-hydrate formate are taken from the literature [10], while we found no records on the crystallographic structure of sodium lactate.

**2θ (°)**	**hkl**	**d_{hkl}** (nm)	**Element**	**Reference**
8.89	0 1 0	0.99	Sodium acetate	00-001-0050
9.05	2 0 0	0.97	Trona	00-011-0643
–	0 0 1	0.75	Sodium formate dihydrate	[11]
–	1 0 0	0.71	Sodium formate dihydrate	[11]
18.11	4 0 0	0.49	Trona	00-011-0643
19.20	0 1 1	0.46	Sodium acetate	00-001-0050
–	2 0 -1	0.38	Sodium formate dihydrate	[11]
–	2 0 0	0.36	Sodium formate dihydrate	[11]
27.10	1 -1 -1	0.33	Trona	00-011-0643
27.55	1 1 0	0.32	Sodium acetate	00-001-0050
29.08	3 1 0	0.31	Trona	00-011-0643
30.47	1 -1 -2	0.29	Nahcolite	00-001-0909
32.29	0 3 1	0.27	Sodium acetate	00-001-0050
–	2 0 1	0.28	Sodium formate dihydrate	[11]
33.87	5 -1 -1	0.26	Trona	00-011-0643
–	3 0 0	0.23	Sodium formate dihydrate	[11]

38.26	1 1 1	0.23	Gold	00-001-1172
–	1 0 -4	0.20	Sodium formate dihydrate	[11]
44.46	0 0 2	0.20	Gold	00-001-1172
47.78	1 -4 -2	0.19	Nahcolite	00-001-0909
51.40	1 3 2	0.17	Nahcolite	00-001-0909
55.34	7 -1 -5	0.16	Trona	00-011-0643
57.82	2 2 2	0.16	Trona	00-011-0643
64.74	2 2 0	0.14	Gold	00-001-1172
77.71	3 1 1	0.12	Gold	00-001-1172

Table S3 Database used for the identification of the crystal structure of the organic materials obtained by PL-CO_2RR of gold target in water. 2θ is the Bragg angle, hkl are the crystalline planes and d_{hkl} is the interplanar spacing.

S.7. HRTEM

High resolution transmission electron microscopy (HRTEM) and energy dispersive x-ray spectroscopy (EDX) were performed at different high acceleration voltages (80 kV, 100 kV, 200 kV), depending on the specificity of the samples as explained in the main text. Two microscopes were used throughout the research, a JEOL 2100F operated at 100 kV or 200 kV, and a FEI Titan 80-300™ operated at 80 kV. In the latter case, the microscope was also used for high-resolution scanning transmission electron microscopy (HRSTEM) with a high-angle annular dark-field detector (HAADF). Different grids were used for the deposition of the samples, in particular Silicon Nitride grids (Si_3N_4, 8 nm membrane thickness), Holey-Carbon 300-mesh copper grids, and Carbon Flat grids subjected to a soft plasma etching process to enhance the contrast of the carbon based structures. To assess the size distribution of the AuNPs, the grid was carefully positioned over a 40 µL drop of the pristine material diluted to give an optical extinction of 0.2. The grid was left in contact with the drop for 60 seconds to remove excess material before being transferred to a 40 µL drop of water for further 60 seconds. Finally, the grid was dried overnight at room temperature. To observe the structure of the photoluminescent organometallic nanocomposites, a 20 µL drop of bleached nanomaterial was deposited directly onto the grid and allowed to dry overnight.

As explained in the main text, some of the organometallic nanocomposites synthesized by pulsed laser driven CO_2RR appeared sensitive to the acceleration voltage of the electrons during transmission electron microscopy (TEM) measurements. For example, in Figures S6(a,b) we show two pictures of the HRTEM of an organometallic nanocomposite taken after

5 s and 15 s of electron irradiation (200 keV), respectively. The interplanar distance associated with the organic material decreases with time leading to complete volatilization and agglomeration of the AuNCs. In Figures S6(c,d) are instead reported the images of organic nanoparticles without gold, similar to those shown in Figure 1(b) of the main text. These nanoparticles rapidly volatilize also under electron irradiation with an acceleration voltage of 100 kV.

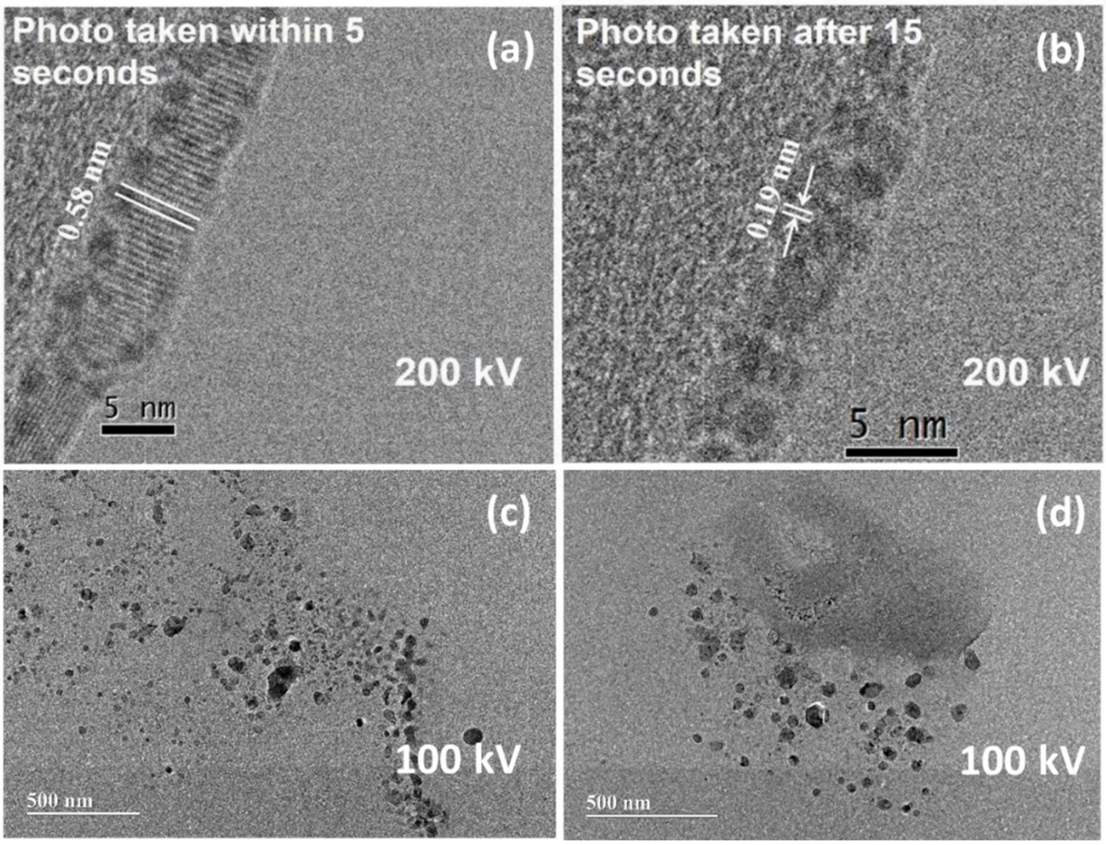

Fig.S6 (a,b) Photographs of the electron microscope screen showing the change in the interplanar distance of a typical gold-acetic ion nanocomposite during the irradiation at the electron acceleration voltage of 200 kV. The electron current was of the order of 10 A/cm^2. (c,d) Organic nanostructures without gold after the bleaching process of the nanomaterial. The nanostructures rapidly volatilize under electron irradiation with an accelerating voltage of 100 kV.

We suspect that the highly volatile gold-free nanoparticles may represent carboxylic acid not complexed with gold. However, it is not possible at this stage to draw any conclusions about the nature of these highly volatile structures, which were also found to be unstable by EDX analysis.

In Figure S7 are reported TEM images at different magnifications of the less volatile photoluminescent nanomaterial after bleaching by centrifugation. Panels (a-d) show the presence of small AuNCs embedded in organic material at low magnification, while in panels (e,f) it is possible to observe some free AuNCs and gold nanoclusters embedded in di-hydrated sodium formate nanocrystals, similarly as reported in Figures 5(e,g) of the main text.

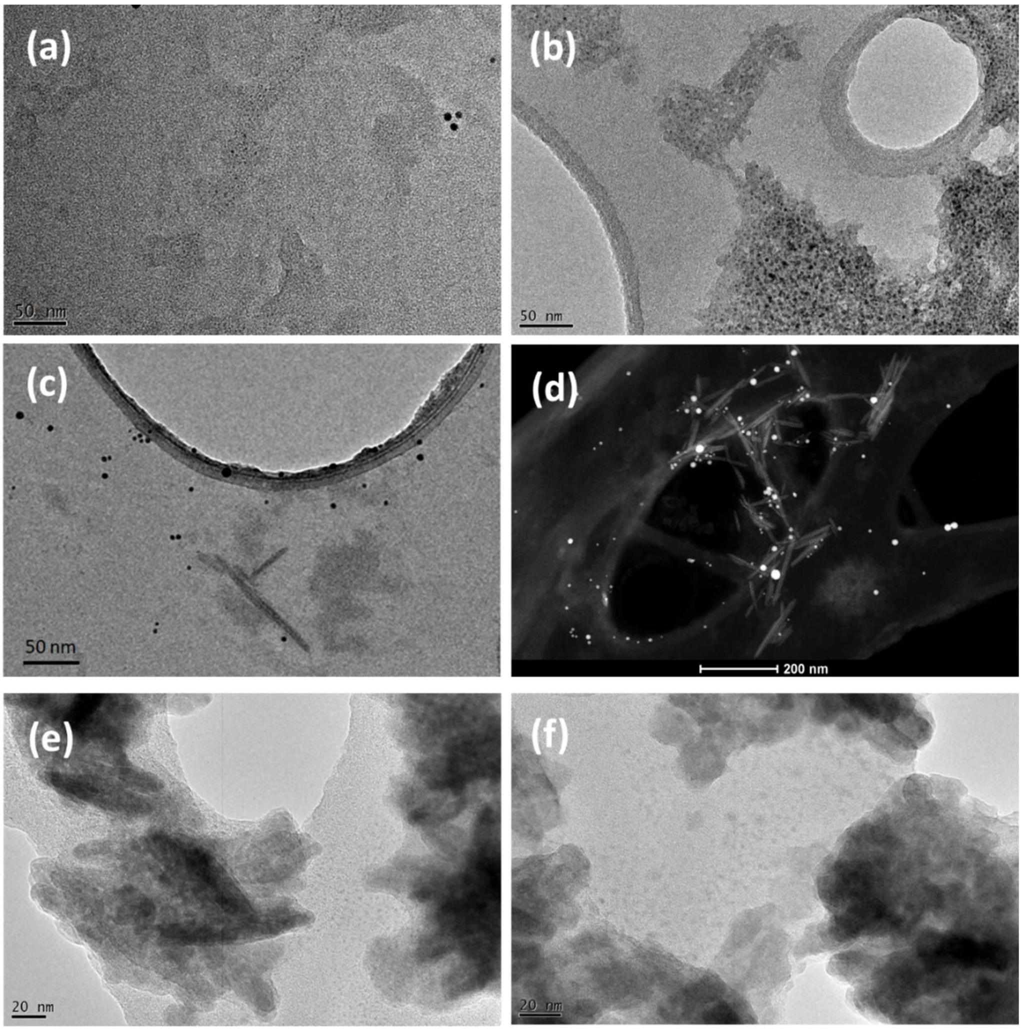

Fig.S7 TEM images of the photoluminescent organometallic nanocomposites obtained after the bleaching process by centrifugation. The samples were synthesized in water in equilibrium with air, containing NaOH at the concentration of 2.0 mmol/L (a,c,e) and 4.0 mmol/L (b,d,f). The nanostructures shown in (e) and (f) were stable even with an electron acceleration voltage of 200 kV, while the needle-like structures of panels (c) and (d) were only stable for 80-100 kV.

To confirm the presence of colloidal structure with dimensions in the order of hundreds of nm prior to the drying process used for the deposition on the TEM grids, we analyzed the samples by Dynamic Light Scattering (DLS) after the bleaching process by centrifugation.

As an example, Figure S8 shows the statistical hydrodynamic size distribution of a sample synthesized by PLAL at the water-air interface with C_{NaOH} = 4.0 mmol/L, using 2ω frequency pulses.

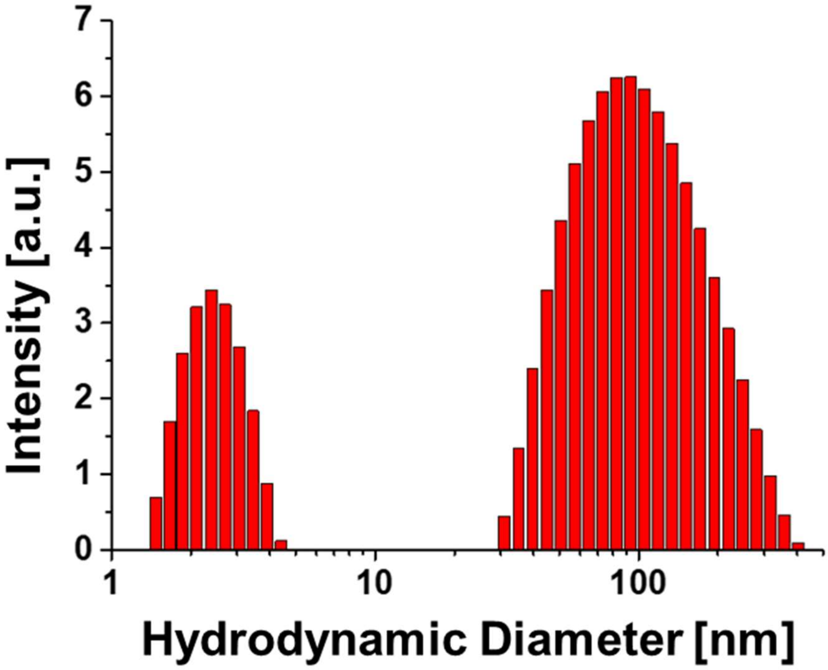

Fig.S8 Statistical hydrodynamic distribution of the nanomaterial present in the sample after bleaching by centrifugation, obtained by DLS. The pristine colloidal dispersion was synthesized by PLAL at the water-air interface (C_{NaOH} = 4.0 mmol/L, 2ω).

S.8. Measurement of the *QY* and decay lifetime of the photoluminescent nanomaterial

Photoluminescence excitation (PLE) and emission (PL) spectra were recorded using a Horiba Jobin Yvon Fluorolog 3-21 spectrofluorometer. A Xenon arc lamp (450 W) was used as the continuous spectrum source, with the excitation wavelength selected by a double Czerny-Turner monochromator, typically in the range 250 - 400 nm. Emission spectra were recorded at room temperature using a single Czerny-Turner monochromator coupled to a thermoelectric cooled R-928 Hamamatsu PMT in the range 350 nm – 550 nm.

Lifetime (LT) measurements were obtained using a NanoLED excitation (wavelength 285 nm or 304 nm, pulse width about 1.2 ns, repetition rate 1 MHz) in Time Correlated Single Photon Counting (TCSPC) configuration, with the same detection system.

To measure the *QY* of the light-emitting species, we used a comparative method with Rhodamine 101 as standard with *QY* = 100% [12]. Both the synthesized and reference samples were diluted to obtain different final concentrations and characterized by UV-Vis absorption and steady-state PL emission. The samples were diluted in the ratios 1:2, 1:4, 1:8, and 1:12 to keep the absorbance less than 0.1. In Figure S9 two typical emission spectra are reported, with excitation at the wavelengths of 304 nm and 530 nm for the synthesized (ω+2ω) and reference samples, respectively.

To calculate the *QY*, the PL emission spectra of the Rhodamine reference sample and the samples synthesized with pulses at the different frequencies (ω, 2ω, ω+2ω), were integrated and plotted as a function of the absorbance (Figure S10(a)) at the corresponding excitation wavelength, as shown in Figure S10(b). The fluorescence quantum yield of the sample (QY_S) is obtained by the following formula:

$$QY_S = \frac{s_S}{s_R} \cdot \left(\frac{n_S}{n_R}\right)^2 \cdot QY_R$$

from the product of the quantum yield of the reference (QY_R = 100%) and the quotient of the slopes of the two linear interpolations (s_S and s_R for sample and reference respectively) multiplied by the squared rate of the refractive indices $(n_S/n_R)^2$.

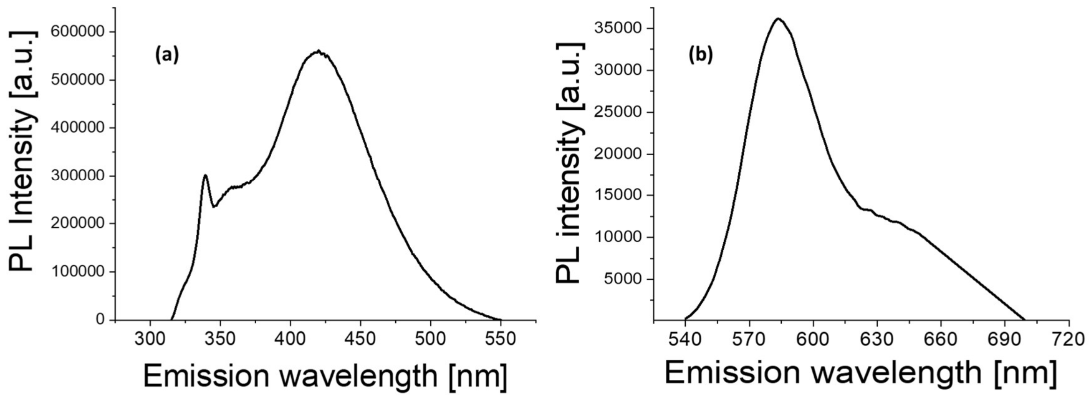

Fig.S9 Example of the spectral window considered for the integration of the PL emission used for the determination of the *QY*: (a) emitting species excited at the wavelength of 304 nm and synthesized by PLAL at the water-air interface, with C_{NaOH} = 4.0 mmol/L, and simultaneous laser pulses at ω and 2ω frequencies; (b) Rhodamine 101, used as standard reference with QY=100%, excited at the wavelength of 530 nm.

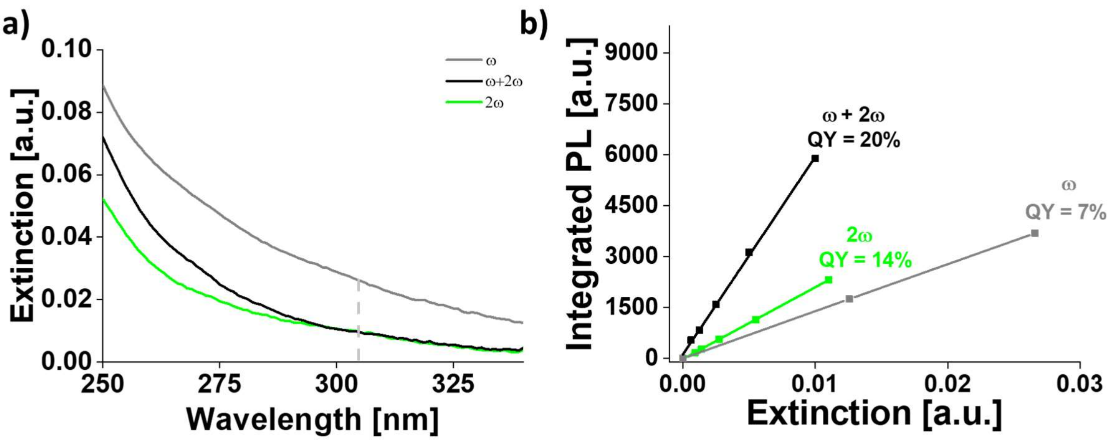

Fig.S10 (a) Typical absorption spectra of the photoluminescent nanomaterial synthesized with pulses at different frequencies, after the bleaching process by concentration and centrifugation, without dilution. The dashed grey line is located at 304 nm, which corresponds to the excitation wavelength of the photoluminescence spectra used to evaluate the *QY* (b) Example of some of the curves used to calculate the *QY* of the samples: integrated photoluminescence in function of the extinction at the value of 304 nm, with linear fit of the experimental points (R^2 ~0.98). The samples were synthesized by PLAL in water at equilibrium with air, with C_{NaOH} = 4.0 mmol/L.

The lifetime of the emitting species was determined by fitting each TRPL decay curve with a double exponential mathematical model, $I = I_1 e^{-(t/\tau_1)} + I_2\, e^{-(t/\tau_2)}$.
Figure S11 shows a representative decay curve with 304 nm excitation and 420 nm emission. The fitted exponents and the obtained parameters are reported as inset. The results of the lifetime in TRPL spectroscopy with variable emission wavelength, are reported in Table S5, and are characteristic of a sample synthesized by PLAL at the water-air interface with C_{NaOH} = 4.0 mmol/L, and simultaneous laser pulses at ω and 2ω frequencies.

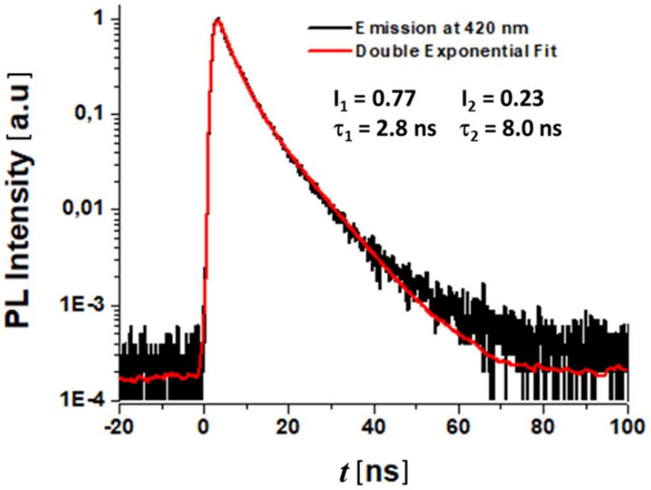

Fig.S11 Typical TRPL decay curve of the emitting species synthesized by PLAL at the water-air interface with C_{NaOH} = 4.0 mmol/L, and simultaneous laser pulses at ω and 2ω frequencies. In this case, the emission was analyzed at the wavelength of 420 nm, with excitation at 304 nm.

Emission [nm]	I_1 [a.u]	T_1 [ns]	I_2 [a.u]	T_2 [ns]
360	0.84	1.44	0.16	8.36
380	0.82	1.73	0.18	7.80
400	0.78	2.28	0.22	7.50
420	0.77	2.79	0.23	7.98
440	0.77	3.02	0.23	8.30
460	0.75	3.04	0.25	8.48

Table S5. Decay parameters as a function of the emission wavelength for the double exponential fit. The emitting species were synthesized by PLAL in water at equilibrium with air, with C_{NaOH} = 4.0 mmol/L, and using simultaneous pulses at ω and 2ω frequencies.

S.9. Viability tests on various normal cell lines

Human microvascular endothelial cells (HMVECs), murine macrophages (RAW 267.4), and human keratinocytes (NCTC 2544) were seeded on 6 cm dishes at a density of 2×10^5 cells in a humidified atmosphere containing 5% CO_2 and then incubated in the appropriate culture media (DMEM for NCTC 2544 and RAW 267.4; EGM2 for HMVECs) (3 mL per well) containing AuNPs at 15 µg/mL for 24 h. Cell cytotoxicity was determined by trypan blue staining: 20 µL of cell suspensions were resuspended with an equal volume of 0.4% (w/v) trypan blue solution prepared in 0.81% NaCl and 0.06% (w/v) dibasic potassium phosphate. Viable and non-viable cells (trypan blue positive) were counted separately using a two-chamber hemocytometer and light microscopy. Light microscopy was used to assess the qualitative intracellular uptake of AuNPs. Cell images were acquired using an EVOS xl core microscope (AMG, Advanced Microscopy Group).

In Figure S12 are reported the viability test performed on human microvascular endothelial cells (HMVECs), murine macrophages (RAW 267.4), and human keratinocytes (NCTC 2544) after incubation with $AuNPs_{Air}$ at a concentration of 15 ppm for 24 hours. The test showed that these nanoparticles were non-toxic at this concentration and were actively taken

up by all the cells tested, resulting in the appearance of black aggregates that could be easily visualized using a conventional optical microscope, as shown in panel (a).

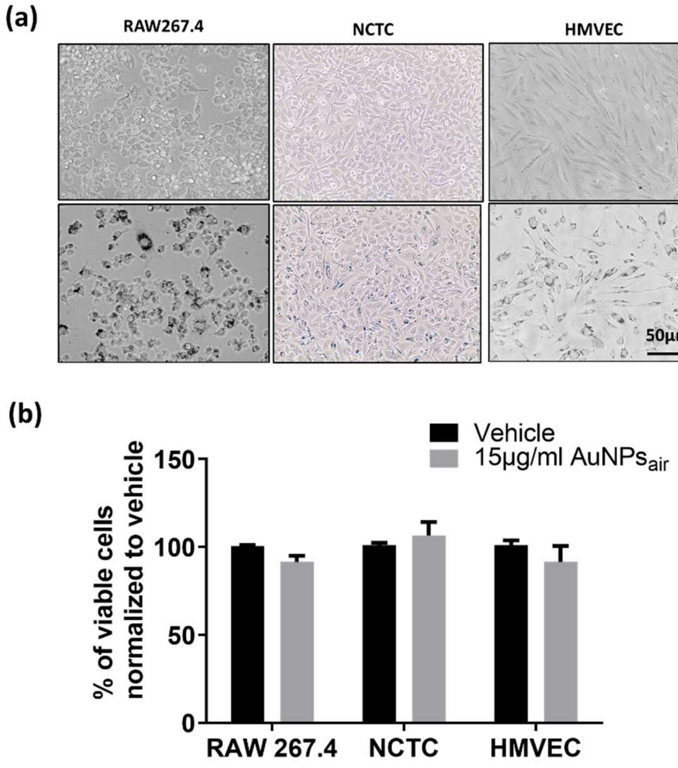

Fig.S12 (a) Light microscopy images of HMVECs, RAW267.4 and NCTC 2544 cell lines enriched with the vehicle (top panel) and with $AuNPs_{Air}$ for 24 hours (bottom panel). (b) Viability results based on trypan blue staining.

References

[1] A. Hornberg, "Propagation of Gaussian beams – A comprehensive introduction," *Laser Technik Journal*, vol. 2, no. 2, pp. 75–80, Jun. 2005, doi: 10.1002/latj.200790044.

[2] S. Dittrich, R. Streubel, C. McDonnell, H. P. Huber, S. Barcikowski, and B. Gökce, "Comparison of the productivity and ablation efficiency of different laser classes for laser ablation of gold in water and air," *Applied Physics A*, vol. 125, no. 6, p. 432, Jun. 2019, doi: 10.1007/s00339-019-2704-8.

[3] S. V. Starinskiy, Y. G. Shukhov, and A. V. Bulgakov, "Laser-induced damage thresholds of gold, silver and their alloys in air and water," *Appl Surf Sci*, vol. 396, pp. 1765–1774, Feb. 2017, doi: 10.1016/j.apsusc.2016.11.221.

[4] S. Dittrich, S. Barcikowski, and B. Gökce, “Plasma and nanoparticle shielding during pulsed laser ablation in liquids cause ablation efficiency decrease,” *Opto-Electronic Advances*, vol. 4, no. 1, pp. 200072–200072, 2021, doi: 10.29026/oea.2021.200072.

[5] F. Giammanco, E. Giorgetti, P. Marsili, and A. Giusti, “Experimental and Theoretical Analysis of Photofragmentation of Au Nanoparticles by Picosecond Laser Radiation,” *The Journal of Physical Chemistry C*, vol. 114, no. 8, pp. 3354–3363, Mar. 2010, doi: 10.1021/jp908964t.

[6] A. R. Ziefuss *et al.*, “Photoluminescence of Fully Inorganic Colloidal Gold Nanocluster and Their Manipulation Using Surface Charge Effects,” *Advanced Materials*, vol. 33, no. 31, Aug. 2021, doi: 10.1002/adma.202101549.

[7] L. B. Scaffardi, N. Pellegri, O. de Sanctis, and J. O. Tocho, “Sizing gold nanoparticles by optical extinction spectroscopy,” *Nanotechnology*, vol. 16, no. 1, pp. 158–163, Jan. 2005, doi: 10.1088/0957-4484/16/1/030.

[8] F. Mafuné, J. Kohno, Y. Takeda, and T. Kondow, “Dissociation and Aggregation of Gold Nanoparticles under Laser Irradiation,” *J Phys Chem B*, vol. 105, no. 38, pp. 9050–9056, Sep. 2001, doi: 10.1021/jp0111620.

[9] R. Jenkins, T. G. Fawcett, D. K. Smith, J. W. Visser, M. C. Morris, and L. K. Frevel, “JCPDS — International Centre for Diffraction Data Sample Preparation Methods in X-Ray Powder Diffraction,” *Powder Diffr*, vol. 1, no. 2, pp. 51–63, Jun. 1986, doi: 10.1017/S0885715600011581.

[10] K. Müller, K.-J. Range, and A. M. Heyns, “Alkalimetallformiate, V Die Kristallstruktur von Natriumformiat-Dihydrat, $NaHCO_2 \cdot 2H_2O$ [1] Alkali Metal Formates, V The Crystal Structure of Sodium Formate Dihydrate, $NaHCO_2 \cdot 2H_2O$ [1],” *Zeitschrift für Naturforschung B*, vol. 49, no. 9, pp. 1179–1182, Sep. 1994, doi: 10.1515/znb-1994-0905.

[11] M. Walker, C. A. Morrison, D. R. Allan, C. R. Pulham, and W. G. Marshall, “A new high pressure phase of sodium formate dihydrate; an experimental and computational study,” *Dalton Transactions*, no. 20, p. 2014, 2007, doi: 10.1039/b613444k.

[12] T. Karstens and K. Kobs, “Rhodamine B and rhodamine 101 as reference substances for fluorescence quantum yield measurements,” *J Phys Chem*, vol. 84, no. 14, pp. 1871–1872, Jul. 1980, doi: 10.1021/j100451a030.
